# Supplementary figures and images for: Levels and functionality of Pacific Islanders’ hybrid humoral immune response to BNT162b2 vaccination and delta/omicron infection: A cohort study in New Caledonia
Source: PLoS Med. 2024 Sep 26;21(9):e1004397. doi: 10.1371/journal.pmed.1004397 (PMC11466435; doi:10.1371/journal.pmed.1004397)

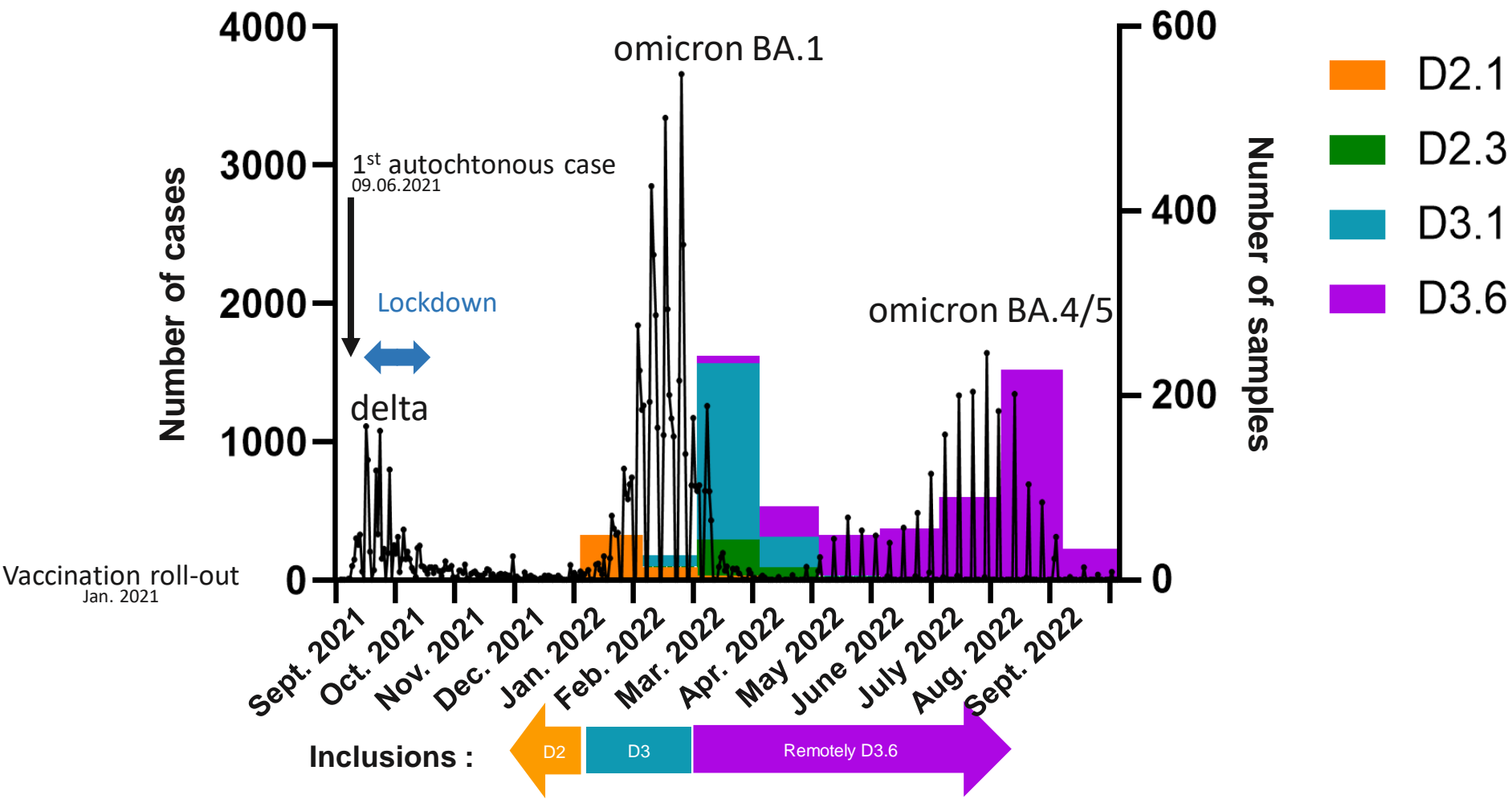

Supplementary figure 1

Supplement: S1 Fig — Absolute number of cases are shown in black on the left y axis, with delta, omicron BA.1 and omicron BA.4/5 epidemic peaks. Absolute number of samples collected 1 month after the second dose (orange), 3 months after the second dose (green), 1 month after the third dose (blue), or 6 months after the third dose (violet) are shown on the right y axis. Inclusion periods are shown below the graph. (PDF) [file pmed.1004397.s001.pdf]

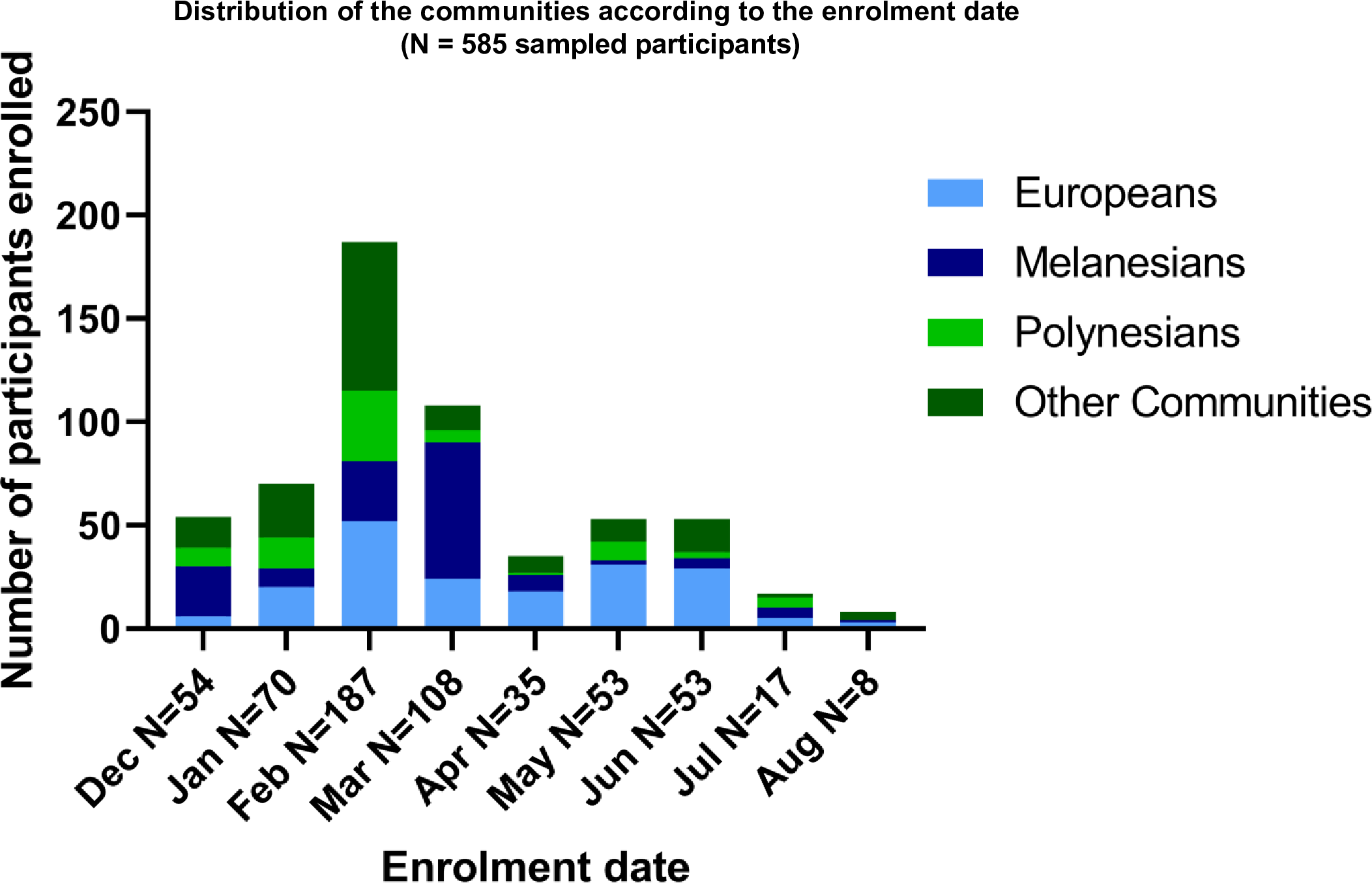

Supplement: S2 Fig — (TIF) [file pmed.1004397.s002.tif]

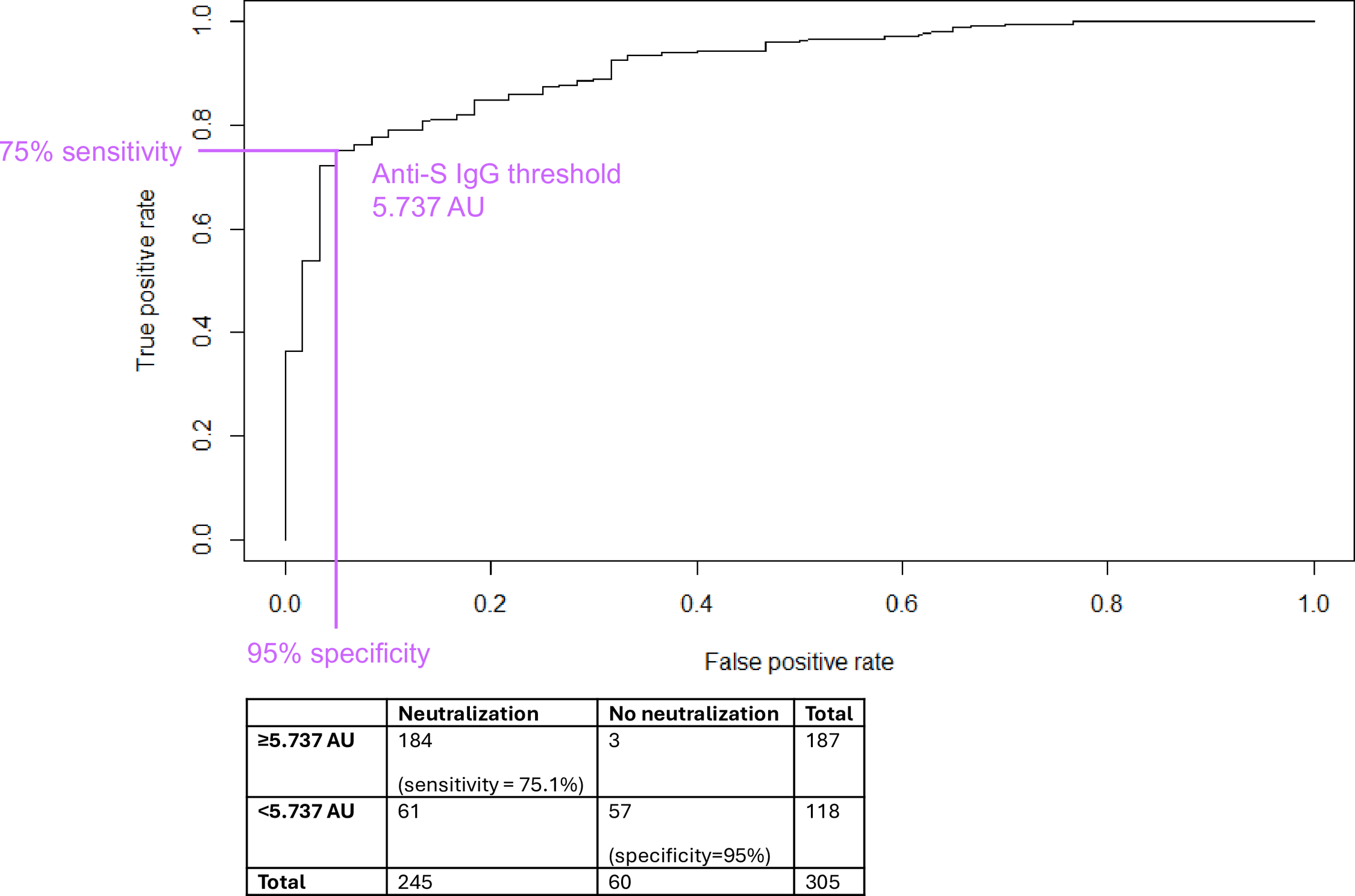

Supplement: S3 Fig — (TIF) [file pmed.1004397.s003.tif]
