# Supplementary material for: Levels and functionality of Pacific Islanders’ hybrid humoral immune response to BNT162b2 vaccination and delta/omicron infection: A cohort study in New Caledonia
Source: PLoS Med. 2024 Sep 26;21(9):e1004397. doi: 10.1371/journal.pmed.1004397 (PMC11466435; doi:10.1371/journal.pmed.1004397)
Supplement: S1 Table — (DOCX) [file pmed.1004397.s004.docx]

**S1 Table. Participants’ description at baseline (1 month after the 2^nd^ or 3^rd^ dose)**

|  | **Total**  **(N=305)** | **Melanesian**  **(N=55)** | **European**  **(N=79)** | **Polynesian**  **(N=55)** | **Other**  **(N=116)** | ***p* value** |
| --- | --- | --- | --- | --- | --- | --- |
| **Women,N (%)** | 167 (54.6) | 31 (56.4) | 43 (54.4) | 37 (67.0) | 56 (48.3) | 0.14* |
| **Age (years)**  **Median (IQR)**  **Range** | 43 (32-55)  19-82 | 41 (28-52)  20-75 | 49 (38-64)  20-81 | 42 (30-50)  19-82 | 41 (31-54)  19-79 | 0.002** |
| **Evaluation time, N (%)**  **Post 2^nd^ dose**  **Post 3^rd^ dose** | 68 (22.2)  237 (77.5) | 22 (40)  33 (60) | 16 (20.2)  63 (79.8) | 9 (16.4)  46 (83.6) | 21 (18.1)  95 (81.9) | 0.006* |
| **Previous infection, N (%)** | 197 (64.6) | 40 (72.7) | 40 (50.6) | 43 (78.2) | 74 (63.8) | 0.005* |
| **Comorbidities, N (%)** | 132 (43.1) | 23 (41.8) | 38 (48.1) | 21 (37.5) | 50 (43.1) | 0.71* |
| **BMI**  **Median**  **(IQR)**  **Range** | 27.8  (23.4-32.4)  15.8-55.4 | 29.8  (26.4-33.1)  18.9-53.6 | 24.8  (21.9-27.9)  15.8-49.1 | 31.5  (27.0-35.9)  21.6-55.4 | 27.9  (22.9-32.4)  17.0-50.6 | <0.001** |

*IQR: inter quartile range; BMI: body mass index*

**Chi-2 test; **Kruskal-Wallis test*
