# Supplementary material for: Levels and functionality of Pacific Islanders’ hybrid humoral immune response to BNT162b2 vaccination and delta/omicron infection: A cohort study in New Caledonia
Source: PLoS Med. 2024 Sep 26;21(9):e1004397. doi: 10.1371/journal.pmed.1004397 (PMC11466435; doi:10.1371/journal.pmed.1004397)
Supplement: S2 Table — (DOCX) [file pmed.1004397.s005.docx]

**S2 Table. Comparison of immune characteristics after the second or third dose**

|  | **n** | **Statistics** | ***p* value** |
| --- | --- | --- | --- |
| **In non-infected participants (n=108)** | | | |
| **Level of anti-S antibodies, median (IQR)**  **Post 2^nd^ dose**  **Post 3^rd^ dose** | 21  87 | 4.94 (4.31, 5.35)  6.05 (5.51, 6.65) | <0.001* |
| **Omicron neutralization≥90%, n (%)**  **Post 2^nd^ dose**  **Post 3^rd^ dose** | 21  87 | 0 (0)  84 (96.6) | <0.001** |
| **Level of CD16 activation, median (IQR)**  **Post 2^nd^ dose**  **Post 3^rd^ dose** | 21  87 | 0.28 (0.13, 1.13)  0.96 (0.60, 1.62) | 0.003* |
| **In infected participants (n=197)** | | | |
| **Level of anti-S antibodies, median (IQR)**  **Post 2^nd^ dose**  **Post 3^rd^ dose** | 47  150 | 5.19 (4.67, 5.81)  6.38 (5.88, 6.84) | <0.001* |
| **Omicron neutralization ≥90%, n (%)**  **Post 2^nd^ dose**  **Post 3^rd^ dose** | 47  150 | 13 (27.7)  148 (98.7) | <0.001** |
| **Level of CD16 activation, median (IQR)**  **Post 2^nd^ dose**  **Post 3^rd^ dose** | 45  150 | 1.06 (0.32, 1.73)  1.10 (0.70, 1.90) | 0.12* |
| **All participants (n=305)** | | | |
| **Level of anti-S antibodies, median (IQR)**  **Post 2^nd^ dose**  **Post 3^rd^ dose** | 68  237 | 5.10 (4.52, 5.64)  6.30 (5.71, 6.80) | <0.001* |
| **Omicron neutralization ≥90%, n (%)**  **Post 2^nd^ dose**  **Post 3^rd^ dose** | 68  237 | 13 (19.1)  232 (97.9) | <0.001** |
| **Level of CD16 activation, median (IQR)**  **Post 2^nd^ dose**  **Post 3^rd^ dose** | 66  237 | 0.70 (0.22, 1.63)  1.05 (0.69, 1.82) | 0.004* |

*IQR: inter quartile range.*

**Wilcoxon test, **Khi-2 test.*
