# Supplementary material for: Levels and functionality of Pacific Islanders’ hybrid humoral immune response to BNT162b2 vaccination and delta/omicron infection: A cohort study in New Caledonia
Source: PLoS Med. 2024 Sep 26;21(9):e1004397. doi: 10.1371/journal.pmed.1004397 (PMC11466435; doi:10.1371/journal.pmed.1004397)
Supplement: S3 Table — (DOCX) [file pmed.1004397.s006.docx]

**S3 Table. Factors associated with anti-S IgG levels one month after immunization, not considering participants from “Other communities” (linear regression)**

|  | **N=189** | **Crude effect (95% CI)** | ***p* value** | **Adjusted effect (95% CI)**  **All variables** | ***p* value** | **Adjusted effect (95% CI)**  **Backward stepwise** | ***p* value** |
| --- | --- | --- | --- | --- | --- | --- | --- |
| **Timepoint**  **Post 2^nd^ dose**  **Post 3^rd^ dose** | 47  142 | *Reference*  **+1.26 (0.98, 1.54)** | **<0.001** | *Reference*  **+1.32 (1.05, 1.59)** | **<0.001** | *Reference*  **+1.35 (1.08, 1.61)** | **<0.001** |
| **Infected**  **No**  **Yes** | 66  123 | *Reference*  +0.27 (-0.03, 0.58) | 0.073 | *Reference*  **+0.26 (0.01, 0.51)** | **0.043** |  |  |
| **Gender***  **Male**  **Female** | 78  111 | **-0.25 (-0.49, 0.00)**  *Reference* | **0.049** | -0.23 (-0.46, 0.01)  *Reference* | 0.06 | *Reference*  ***-0.24 (-0.47, 0.00)*** | **0.048** |
| **Age (years)***  **18-39**  **40-64**  **≥65** | 75  83  31 | *Reference*  **-0.27 (-0.54, -0.01)**  **-0.46 (-0.82, -0.11)** | **0.019** | *Reference*  -0.26 (-0.54, 0.02)  -0.42 (-0.82, -0.02) | 0.072 | *Reference*  **-0.27 (-0.52, -0.01)**  **-0.44 (-0.79, -0.09)** | **0.023** |
| **Comorbidities***  **No**  **Yes** | 107  82 | *Reference*  -0.08 (-0.33, 0.16) | 0.5 | *Reference*  0.02 (-0.25, 0.28) | 0.90 |  |  |
| **BMI***  **Underweight**  **Normal**  **Overweight**  **Obese** | 3  55  59  72 | -1.17 (-2.12, -0.22)  *Reference*  -0.01 (-0.31, 0.29)  +0.39 (0.10, 0.67) | **<0.001** | **-1.22 (-2.16, -0.28)**  *Reference*  +0.10 (-0.22, 0.41)  **+0.44 (0.11, 0.76)** | **<0.001** | **-1.26 (-2.20, -0.32)**  *Reference*  +0.10 (-0.21, 0.40)  **+0.39 (+0.11, 0.68)** | **<0.001** |
| **Community***  **European**  **Melanesian**  **Polynesian** | 79  55  55 | *Reference*  +0.10 (-0.20, 0.40)  +0.34 (0.05, 0.63) | 0.067 | *Reference*  -0.18 (-0.49, 0.13)  -0.07 (-0.39, 0.26) | 0.49 |  |  |

**the univariate analysis is adjusted for timepoint.*

*CI: confidence interval; BMI: body mass index.*

*BMI classes: Underweight = BMI<18.5 kg/m², Normal weight = BMI є [18.5, 25[ kg/m², Overweight = BMI є [25, 30[ kg/m², Obese = BMI ≥30 kg/m².*
