# Supplementary material for: Levels and functionality of Pacific Islanders’ hybrid humoral immune response to BNT162b2 vaccination and delta/omicron infection: A cohort study in New Caledonia
Source: PLoS Med. 2024 Sep 26;21(9):e1004397. doi: 10.1371/journal.pmed.1004397 (PMC11466435; doi:10.1371/journal.pmed.1004397)
Supplement: S4 Table — (DOCX) [file pmed.1004397.s007.docx]

**S4 Table. Factors associated with the ability to neutralize Omicron BA.1 and/or BA.4-5 one month after the second and third dose, not considering participants from “Other communities” (logistic regression)**

| ***One month after the second dose*** | | | | | | |
| --- | --- | --- | --- | --- | --- | --- |
|  | **N=47** | **Neutralisation, n (%)** | **Crude OR (95% CI)** | ***p* value** | **Adjusted OR (95% CI)**  **All variables** | ***p* value** |
| **Infected***  **No**  **Yes** | 15  32 | 0 (0)  7 (21.9) | 1  9.12 (0.99, 1214.01) | 0.052 | 1  4.48 (0.38, 491.23) | 0.3 |
| **Level of anti-S IgG**  **<5.737 AU**  **≥ 5.737 AU** | 40  7 | 2 (5.00)  5 (71.4) | **1**  **47.50 (6.47, 568.55)** | **<0.001** | 1  6.03 (0.81, 59.35) | 0.079 |
| **Gender**  **Male**  **Female** | 19  28 | 3 (15.8)  4 (14.3) | 1  1.13 (0.20, 5.78) | 0.90 | 0.77 (0.06, 5.94)  1 | 0.8 |
| **Age (years)***  **18-39**  **40-64**  **≥65** | 24  17  6 | 4 (16.7)  3 (17.6)  0 (0) | 1  1.10 (0.22, 5.24)  0.35 (0.00, 4.05) | 0.77 | 1  1.32 (0.04, 30.36)  0.89 (0.00, 40.59) | 0.95 |
| **Comorbidities**  **No**  **Yes** | 27  20 | 4 (14.8)  3 (15.0) | 1  1.01 (0.18, 5.20) | >0.90 | 1  0.80 (0.04, 20.12) | 0.9 |
| **BMI**  **Normal weight**  **Overweight**  **Obese** | 12  17  18 | 1 (8.3)  1 (5.9)  5 (27.8) | 1  0.69 (0.03, 18.64)  4.23 (0.57, 87.64) | 0.19 | 1  1.70 (0.08, 59.32)  3.82 (0.26, 159.38) | 0.52 |
| **Community**  **European**  **Melanesian**  **Polynesian** | 16  22  9 | 3 (18.8)  3 (13.6)  1 (11.1) | 1  0.68 (0.11, 4.20)  0.54 (0.02, 5.11) | 0.86 | 1  0.40 (0.01, 4.51)  0.34 (0.01, 6.63) | 0.66 |
| ***One month after the third dose*** | | | | | | |
|  | **N=142** | **Neutralisation, n (%)** | **Crude OR (95% CI)** | ***p* value** | **Adjusted OR (95% CI)**  **All variables** | ***p* value** |
| **Infected**  **No**  **Yes** | 51  91 | 49 (94.2)  89 (9.8) | 1  1.82 (0.21, 15.5) | 0.60 | 1  0.94 (0.08, 7.59) | 0.90 |
| **Level of anti-S IgG**  **<5.737 AU**  **≥ 5.737 AU** | 37  105 | 34 (89.5)  104 (99.0) | 1  9.18 (1.13, 188.9) | 0.058 | 1  3.54 (0.44, 34.34) | 0.20 |
| **Gender**  **Male**  **Female** | 59  83 | 57 (95.0)  81 (97.6) | 0.70 (0.08, 6.00)  1 | 0.70 | 0.64 (0.06, 5.33)  1 | 0.70 |
| **Age (years)***  **18-39**  **40-64**  **≥65** | 51  66  25 | 51 (100)  63 (95.5)  24 (96.0) | 1  0.18 (0.00, 1.88)  0.16 (0.00, 3.08) | 0.49 | 1  0.26 (0.00, 2.98)  0.14 (0.00, 6.04) | 0.34 |
| **Comorbidities**  **No**  **Yes** | 80  62 | 78 (96.3)  60 (96.8) | 1  0.77 (0.09, 6.56) | 0.80 | 1  1.02 (0.13, 10.02) | 0.90 |
| **BMI***  **Underweight**  **Normal weight**  **Overweight**  **Obese** | 3  43  42  54 | 2 (66.7)  41 (95.3)  42 (100)  53 (89.1) | 0.10 (0.01, 1.39)  1  5.12 (0.40, 714.62)  2.15 (0.28, 24.14) | 0.068 | 0.16 (0.00, 4.15)  1  6.40 (0.50, 880.38)  2.43 (0.19, 41.03) | 0.13 |
| **Community***  **European**  **Melanesian**  **Polynesian** | 63  33  46 | 61 (96.8)  33 (100)  44 (95.7) | 1  2.72 (0.21; 379.95)  0.72 (0.11; 4.85) | 0.69 | 1  1.04 (0.05, 156.45)  0.34 (0.02, 3.70) | 0.43 |

*CI: confidence interval, BMI: body mass index, OR: Odds Ratio, AU: Arbitrary Units.*

**: Logistic regression with Firth’s correction.*

*BMI classes: Underweight = BMI<18.5 kg/m², Normal weight = BMI є [18.5, 25[ kg/m², Overweight = BMI є [25, 30[ kg/m², Obese = BMI ≥30 kg/m².*
