# Supplementary material for: Levels and functionality of Pacific Islanders’ hybrid humoral immune response to BNT162b2 vaccination and delta/omicron infection: A cohort study in New Caledonia
Source: PLoS Med. 2024 Sep 26;21(9):e1004397. doi: 10.1371/journal.pmed.1004397 (PMC11466435; doi:10.1371/journal.pmed.1004397)
Supplement: S5 Table — (DOCX) [file pmed.1004397.s008.docx]

**S5 Table. Factors associated with the level of ADCC (CD16 activation) one month after immunization, not considering participants from “Other communities” (Linear regression)**

|  | **n=187** | **Crude effect (95% CI)** | ***p* value** | **Adjusted effect (95% CI)**  **All variables** | ***p* value** |
| --- | --- | --- | --- | --- | --- |
| **Timepoint**  **Post 2^nd^ dose**  **Post 3^rd^ dose** | 45  142 | *Reference*  +0.41 (-0.11, 0.93) | 0.12 | *Reference*  +0.04 (-0.59, 0.68) | 0.90 |
| **Infected**  **No**  **Yes** | 66  121 | *Reference*  -0.09 (-0.56, 0.38) | 0.70 | *Reference*  -0.03 (-0.53, 0.46) | 0.90 |
| **Level of anti-S IgG**  **<5.737 AU**  **≥ 5.737 AU** | 76  111 | ***Reference***  **+0.50 (0.04, 0.95)** | **0.032** | *Reference*  +0.48 (-0.09, 1.04) | 0.10 |
| **Gender**  **Male**  **Female** | 76  111 | +0.38 (-0.07, 0.84)  *Reference* | 0.10 | +0.37 (-0.09, 0.83)  *Reference* | 0.12 |
| **Age (years)**  **18-39**  **40-64**  **≥65** | 74  82  31 | *Reference*  +0.26 (-0.23, 0.76)  -0.23 (-0.88, 0.43) | 0.28 | *Reference*  +0.20 (-0.34, 0.73)  -0.42 (-1.19, 0.35) | 0.19 |
| **Comorbidities**  **No**  **Yes** | 106  81 | *Reference*  -0.06 (-0.52, 0.39) | 0.80 | *Reference*  -0.06 (-0.58, 0.46) | 0.80 |
| **BMI**  **Underweight**  **Normal**  **Overweight**  **Obese** | 3  55  58  71 | -1.24 (-3.07, 0.58)  *Reference*  -0.10 (-0.68, 0.48)  -0.31 (-0.87, 0.24) | 0.42 | -1.25 (-3.09, 0.58)  *Reference*  +0.17 (-0.44, 0.78)  -0.09 (-0.72, 0.55) | 0.43 |
| **Community**  **European**  **Melanesian**  **Polynesian** | 78  54  55 | ***Reference***  **-0.71 (-1.24, -0.17)**  **-0.36 (-0.90, 0.17)** | **0.033** | *Reference*  -0.64 (-1.24, -0.04)  -0.44 (-1.07, 0.20) | 0.11 |

*CI: confidence interval; BMI: body mass index. AU: Arbitrary Units.*

*BMI classes: Underweight = BMI<18.5 kg/m², Normal weight = BMI є [18.5, 25[ kg/m², Overweight = BMI є [25, 30[ kg/m², Obese = BMI ≥30 kg/m².*

*Following the backward stepwise procedure, no combination of variables remained significantly associated with the level of ADCC.*
