# Supplementary material for: Levels and functionality of Pacific Islanders’ hybrid humoral immune response to BNT162b2 vaccination and delta/omicron infection: A cohort study in New Caledonia
Source: PLoS Med. 2024 Sep 26;21(9):e1004397. doi: 10.1371/journal.pmed.1004397 (PMC11466435; doi:10.1371/journal.pmed.1004397)
Supplement: S6 Table — (DOCX) [file pmed.1004397.s009.docx]

**S6 Table. Description of the participants followed up at month 1 and month 6 post-third dose of vaccination (N=214)**

|  | **Total**  **(N=214)** | **Melanesian**  **(N=29)** | **European**  **(N=57)** | **Polynesian**  **(N=42)** | **Other**  **(N=86)** | ***P* value** |
| --- | --- | --- | --- | --- | --- | --- |
| **Women, N (%)** | 121 (56.5) | 16 (55.2) | 33 (57.9) | 29 (69.0) | 43 (50.0) | 0.24* |
| **Age (Years)**  **Median (IQR)**  **Range** | 45 (36-59)  19-81 | 42 (32-60)  20-75 | 50 (39-64)  20-81 | 43 (36-49)  19-70 | 44 (33-57)  19-79 | 0.022** |
| **Comorbidities, N (%)** | 92 (43) | 13 (44.8) | 26 (45.6) | 16 (38.1) | 37 (43.0) | 0.89* |
| **BMI (kg/m²)**  **Median (IQR)**  **Range** | 27.5 (23.4-32.4)  15.8-55.4 | 31.3 (26.1-33.7)  21.1-53.6 | 25.0 (21.7- 27.5)  15.8-49.1 | 31.4 (26.4- 35.9)  21.6-55.4 | 27.7 (23.0- 32.1)  17.0-49.0 | <0.001** |
| **Previous Infection, N (%)**  **No**  **Yes, at M1 ONLY**  **Yes, between M1 and M6**  **Reinfection between M1 and M6** | 42 (19.6)  59 (27.6)  40 (18.7)  73 (34.1) | 3 (10.4)  9 (31.0)  6 (20.7)  11 (37.9) | 19 (33.3)  16 (28.1)  9 (15.8)  13 (22.8) | 2 (4.8)  14 (33.3)  8 (19.0)  18 (42.9) | 18 (20.9)  20 (23.3)  17 (19.8)  31 (36.0) | 0.059* |
| **Level of anti-S antibodies**  **Median (IQR) at M1**  **Median (IQR) at M6**  **Median (IQR, Range) decrease from M1 to M6** | 6.30 (5.72-6.74)  4.82 (3.68-5.73)  1.67 (0.63-2.59)  -2.17, 4.90 | 6.14 (5.66-6.52)  4.61 (3.19-5.79)  1.58 (0.78-2.63)  -1.51, 3.46 | 6.14 (5.72-6.62)  5.09 (3.10-5.81)  1.42 (0.65-2.66)  -2.17, 4.24 | 6.44 (5.97-6.78)  4.91 (3.92-5.58)  1.82 (0.87-2.43)  -0.90, 4.90 | 6.35 (5.71-6.83)  4.78 (3.70-5.70)  1.65 (0.43-2.65)  -1.65, 4.76 | 0.19**  0.95**  0.96** |
| **Level of ADCC (CD16 activation, N=211)**  **Median (IQR) at M1**  **Median (IQR) at M6**  **Median (IQR, Range) decrease from M1 to M6** | 1.06 (0.69, 1.94)  0.85 (0.34, 1.98)  0.20 (-0.41, 0.61)  -4.74, 4.05 | 0.93 (0.52, 1.37)  0.55 (0.22, 1.46)  0.30 (-0.19, 0.61)  -2.24, 2.30 | 1.35 (0.77, 2.45)  0.83 (0.36, 2.17)  0.25 (-0.48, 0.63)  -4.74, 3.83 | 0.79 (0.47, 1.46)  0.68 (0.29, 1.50)  0.00 (-0.19, 0.51)  -4.72, 1.86 | 1.11 (0.75, 1.84)  1.09 (0.45, 2.40)  0.20 (-0.68, 0.59)  -4.06, 4.05 | 0.05**  0.27**  0.81** |
| **Omicron neutralization >90%, N (%)**  **M1**  **M6** | 209 (97.7)  176 (82.2)† | 29 (100)  25 (86.2) | 55 (96.5)  43 (75.4)† | 40 (95.2)  37 (88.1) | 85 (98.8)  71 (82.6)† | 0.45*  0.38* |

**Khi-2 test; **Kruskal-Wallis test.*

*† two participants acquired the capacity to neutralize Omicron between M1 and M6: one European and one participant belonging to Other communities.*
