# Supplementary material for: Levels and functionality of Pacific Islanders’ hybrid humoral immune response to BNT162b2 vaccination and delta/omicron infection: A cohort study in New Caledonia
Source: PLoS Med. 2024 Sep 26;21(9):e1004397. doi: 10.1371/journal.pmed.1004397 (PMC11466435; doi:10.1371/journal.pmed.1004397)
Supplement: S7 Table — (DOCX) [file pmed.1004397.s010.docx]

**S7 Table. Factors associated with the progression of anti-S IgG levels between one and six months after immunization (linear regression)**

|  | **N=214** | **Crude effect**  **(95% CI)** | ***p* value** | **Adjusted effect**  **(95% CI)**  **All variables** | ***p* value** | **Adjusted effect**  **(95% CI)**  **Backward stepwise** | ***p* value** |
| --- | --- | --- | --- | --- | --- | --- | --- |
| **Previous infection, n (%)**  **No**  **Yes, at M1**  **Yes, between M1 and M6**  **Reinfection between M1 and M6** | 42  59  40  73 | *Reference*  -0.04 (-0.50, 0.43)  **+1.73 (1.22, 2.24)**  **+1.17 (0.73, 1.62)** | **<0.001** | ***Reference***  +0.22 (-0.26, 0.70)  **+1.88 (1.35, 2.40)**  **+1.43 (0.96, 1.90)** | **<0.001** | *Reference**  +0.08 (-0.38, 0.55)  **+1.77 (1.27, 2.28)**  **+1.29 (0.84, 1.74)** | **<0.001** |
| **Gender**  **Female**  **Male** | 121  93 | *Reference*  +0.01 (-0.37, 0.38) | 0.99 | *Reference*  0.04 (-0.29, 0.37) | 0.80 |  |  |
| **Age (years)**  **18-39**  **40-64**  **≥65** | 76  106  32 | *Reference*  +0.12 (-0.29, 0.53)  -0.13 (-0.70, 0.44) | 0.64 | *Reference*  +0.06 (-0.30, 0.41)  -0.21 (-0.75, 0.34) | 0.58 |  |  |
| **Comorbidities**  **No**  **Yes** | 122  92 | *Reference*  -0.05 (-0.42, 0.32) | 0.80 | *Reference*  +0.02 (-0.33, 0.37) | 0.90 |  |  |
| **BMI**  **Underweight**  **Normal**  **Overweight**  **Obese** | 7  66  61  80 | -0.14 (-1.21, 0.93)  *Reference*  +0.38 (-0.10, 0.86)  -0.20 (-0.65, 0.24) | 0.088 | -0.27 (-1.21, 0.66)  *Reference*  +0.38 (-0.04, 0.80)  +0.06 (-0.36, 0.48) | 0.22 |  |  |
| **Community**  **European**  **Melanesian**  **Polynesian**  **Other** | 57  29  42  86 | *Reference*  -0.04 (-0.66, 0.58)  -0.21 (-0.76, 0.35)  -0.03 (-0.49, 0.44) | 0.89 | *Reference*  -0.37 (-0.93, 0.18)  -0.55 (-1.07, -0.03)  -0.28 (-0.68, 0.13) | 0.21 |  |  |
| **Level of anti-S IgG at M1**  **<5.737 AU**  **≥ 5.737 AU** | 55  159 | *Reference*  **-0.50 (-0.92, -0.08)** | **0.019** | ***Reference***  **-0.50 (-0.88, -0.12)** | **0.010** | *Reference**  **-0.54 (-0.91, -0.18)** | **0.004** |
| **Neutralization at M1**  **No**  **Yes** | 5  209 | *Reference*  -0.79 (-2.01, 0.43) | 0.20 | *Reference*  -0.02 (-1.12, 1.08) | 0.90 |  |  |

*CI: confidence interval; BMI: body mass index.*

*BMI classes: Underweight = BMI<18.5 kg/m², Normal weight = BMI є [18.5, 25[ kg/m², Overweight = BMI є [25, 30[ kg/m², Obese = BMI ≥30 kg/m².*

**The mean difference between D3.1 and D3.6 in participants with no previous infection and with anti-S IgGs level <5.737 at M1 was -1.98 (-2.39; -1.56).*
