# Supplementary material for: Levels and functionality of Pacific Islanders’ hybrid humoral immune response to BNT162b2 vaccination and delta/omicron infection: A cohort study in New Caledonia
Source: PLoS Med. 2024 Sep 26;21(9):e1004397. doi: 10.1371/journal.pmed.1004397 (PMC11466435; doi:10.1371/journal.pmed.1004397)
Supplement: S8 Table — (DOCX) [file pmed.1004397.s011.docx]

**S8 Table. Impact of ethnicity on the levels of anti-N IgG at one-month post-immunization in infected individuals (linear regression)**

|  | **N=197** | **Crude effect (95% CI)** | ***p* value** |
| --- | --- | --- | --- |
| **Community**  **European**  **Melanesian**  **Polynesian**  **Other** | 40  40  43  74 | Reference  0.45 (0.10, 0.80)  0.38 (0.04, 0.72)  0.24 (-0.07, 0.55) | 0.056 |

*CI: confidence interval.*
