# Supplementary material for: Levels and functionality of Pacific Islanders’ hybrid humoral immune response to BNT162b2 vaccination and delta/omicron infection: A cohort study in New Caledonia
Source: PLoS Med. 2024 Sep 26;21(9):e1004397. doi: 10.1371/journal.pmed.1004397 (PMC11466435; doi:10.1371/journal.pmed.1004397)
Supplement: S9 Table — (DOCX) [file pmed.1004397.s012.docx]

**S9 Table. Impact of ethnicity on the decrease in the levels of anti-N IgG between one and six months post-third dose in individuals infected at one month post-third dose (linear regression)**

|  | **N=59** | **Crude effect (95% CI)** | ***p* value** |
| --- | --- | --- | --- |
| **Community**  **European**  **Melanesian**  **Polynesian**  **Other** | 16  9  14  20 | Reference  **-1.01 (-1.59, -0.44)**  -0.43 (-0.94, 0.07)  -0.30 (-0.77, 0.16) | **0.005** |

*CI: confidence interval.*
