# Supplementary material for: Levels and functionality of Pacific Islanders’ hybrid humoral immune response to BNT162b2 vaccination and delta/omicron infection: A cohort study in New Caledonia
Source: PLoS Med. 2024 Sep 26;21(9):e1004397. doi: 10.1371/journal.pmed.1004397 (PMC11466435; doi:10.1371/journal.pmed.1004397)
Supplement: S10 Table — (DOCX) [file pmed.1004397.s013.docx]

**S10 Table. Factors associated with the loss of the ability to neutralize Omicron BA.1 and/or BA.4-5 between one and six months after immunization (logistic regression)**

|  | **N=209** | **Loss of neutralization**  **N (%)** | **Crude OR**  **(95% CI)** | ***p* value** | **Adjusted OR**  **(95% CI)**  **All variables** | ***p* value** | **Adjusted OR**  **(95% CI)**  **Backward stepwise** | ***p* value** |
| --- | --- | --- | --- | --- | --- | --- | --- | --- |
| **Previous Infection, N (%)**  **No**  **Yes, at M1 only**  **Yes, between M1 and M6**  **Reinfection between M1 and M6** | 42  58  37  72 | **20 (47.6)**  **10 (17.2)**  **2 (5.4)**  **3 (4.2)** | 1  **0.23 (0.09, 0.56)**  **0.06 (0.01, 0.24)**  **0.05 (0.01, 0.16)** | **<0.001** | 1  **0.19 (0.06, 0.55)**  **0.06 (0.01, 0.26)**  **0.03 (0.01, 0.14)** | **<0.001** | 1  **0.20 (0.07, 0.53)**  **0.06 (0.01, 0.24)**  **0.04 (0.01, 0.15)** | **<0.001** |
| **Gender**  **Female**  **Male** | 119  90 | 14 (11.8)  21 (23.3) | 1  **2.28 (1.10, 4.88)** | **0.029** | **1**  **2.96 (1.16, 8.03)** | **0.026** | **1**  **2.64 (1.12, 6.56)** | **0.030** |
| **Age (years)**  **18-39**  **40-64**  **≥65** | 76  102  31 | **4 (5.3)**  **22 (21.6)**  **9 (29.0)** | **1**  **4.95 (1.79, 17.53)**  **7.36 (2.18, 29.38)** | **0.006** | **1**  **7.01 (2.08, 29.95)**  **10.01 (2.12, 57.21)** | **0.008** | **1**  **5.74 (1.87, 22.19)**  **7.89 (2.02, 36.13)** | **0.008** |
| **Comorbidities**  **No**  **Yes** | 120  89 | 15 (12.5)  20 (22.5) | 1  2.03 (0.98, 4.29) | 0.059 | 1  1.81 (0.66, 5.10) | 0.30 |  |  |
| **BMI**  **Underweight**  **Normal**  **Overweight**  **Obese** | 6  64  60  79 | **1 (16.7)**  **12 (18.8)**  **8 (13.3)**  **14 (17.7)** | 0.87 (0.04, 6.06)  1  0.67 (0.24, 1.75)  0.93 (0.40, 2.22) | 0.87 | 0.57 (0.02, 10.28)  1  0.25 (0.06, 0.89)  0.69 (0.21, 2.30) | 0.21 |  |  |
| **Community**  **European**  **Melanesian**  **Polynesian**  **Other** | 55  29  40  85 | 13 (23.6)  4 (13.8)  3 (7.5)  15 (17.6) | 1  0.52 (0.13, 1.65)  0.26 (0.06, 0.89)  0.69 (0.30, 1.61) | 0.23 | 1  1.70 (0.32, 8.55)  0.74 (0.11, 4.19)  1.39 (0.47, 4.31) | 0.78 |  |  |
| **Level of anti-S IgG at M1**  **<5.737 AU**  **≥ 5.737 AU** | 51  158 | 11 (21.6)  24 (15.2) | 1  0.65 (0.30, 1.49) | 0.30 | 1  0.70 (0.26, 1.97) | 0.50 |  |  |

*CI: confidence interval; BMI: body mass index, OR: Odds Ratio.*

*BMI classes: Underweight = BMI<18.5 kg/m², Normal weight = BMI є [18.5, 25[ kg/m², Overweight = BMI є [25, 30[ kg/m², Obese = BMI ≥30 kg/m².*
