# Supplementary material for: Levels and functionality of Pacific Islanders’ hybrid humoral immune response to BNT162b2 vaccination and delta/omicron infection: A cohort study in New Caledonia
Source: PLoS Med. 2024 Sep 26;21(9):e1004397. doi: 10.1371/journal.pmed.1004397 (PMC11466435; doi:10.1371/journal.pmed.1004397)
Supplement: S11 Table — (DOCX) [file pmed.1004397.s014.docx]

**S11 Table. Factors associated with the variation in the level of ADCC (CD16 activation in response to SARS-CoV-2 Spike protein from the ancestral strain Wuhan) between one and six months after immunization (linear regression)**

|  | **N=211** | **Crude effect**  **(95% CI)** | ***p* value** | **Adjusted effect**  **(95% CI)**  **All variables** | ***p* value** | **Adjusted effect**  **(95% CI)**  **Backward stepwise** | ***p* value** |
| --- | --- | --- | --- | --- | --- | --- | --- |
| **Previous Infection, N (%)**  **No**  **Yes, at M1**  **Yes, between M1 and M6**  **Reinfection between M1 and M6** | 41  58  39  73 | *Reference*  -0.12 (-0.57, 0.33)  **+0.67 (0.17, 1.16)**  +0.41 (-0.03, 0.84) | **0.002** | ***Reference***  **-0.12 (-0.59, 0.36)**  **+0.68 (0.17, 1.19)**  **+0.34 (-0.12, 0.80)** | **0.004** | *Reference**  -0.10 (-0.55, 0.35)  **0.66 (0.17, 1.16)**  0.39 (-0.04, 0.82) | **0.003** |
| **Gender**  **Female**  **Male** | 120  91 | *Reference*  -0.35 (-0.66, -0.03) | **0.030** | *Reference*  **-0.32 (-0.64, 0.00)** | **0.050** | *Reference**  **-0.31 (-0.62, 0.00)** | **0.049** |
| **Age (years)**  **18-39**  **40-64**  **≥65** | 76  104  31 | *Reference*  0.27 (-0.07, 0.62)  -0.26 (-0.74, 0.22) | **0.047** | *Reference*  +0.31 (-0.04, 0.66)  -0.16 (-0.70, 0.38) | 0.066 |  |  |
| **Comorbidities**  **No**  **Yes** | 121  90 | *Reference*  0.03 (-0.28, 0.35) | 0.80 | *Reference*  +0.04 (-0.30, 0.39) | 0.80 |  |  |
| **BMI**  **Underweight**  **Normal**  **Overweight**  **Obese** | 6  65  61  79 | 0.31 (-0.67, 1.29)  *Reference*  0.07 (-0.34, 0.48)  0.11 (-0.28, 0.49) | 0.90 | -0.03 (-1.00, 0.93)  *Reference*  +0.09 (-0.32, 0.51)  +0.13 (-0.28, 0.55) | 0.93 |  |  |
| **Community**  **European**  **Melanesian**  **Polynesian**  **Other** | 56  29  41  85 | *Reference*  0.01(-0.52, 0.53)  0.25 (-0.22, 0.72)  0.17 (-0.22, 0.57) | 0.67 | *Reference*  -0.07 (-0.61, 0.47)  +0.01 (-0.50, 0.53)  +0.09 (-0.31, 0.49) | 0.92 |  |  |
| **Level of anti-S IgG at M1**  **<5.737 AU**  **≥ 5.737 AU** | 52  159 | *Reference*  0.24 (-0.12, 0.61) | 0.20 | *Reference*  +0.25 (-0.13, 0.62) | 0.20 |  |  |
| **Neutralization at M1**  **No**  **Yes** | 2  209 | *Reference*  0.10 (-1.23, 1.42) | 0.90 | *Reference*  +0.46 (-0.87, 1.79) | 0.50 |  |  |

*CI: confidence interval; BMI: body mass index.*

*BMI classes: Underweight = BMI<18.5 kg/m², Normal weight = BMI є [18.5, 25[ kg/m², Overweight = BMI є [25, 30[ kg/m², Obese = BMI ≥30 kg/m².*

**The mean difference between D3.1 and D3.6 in participants with no previous infection and of female gender was -0.16 (-0.53; 0.21).*
