# Supplementary material for: Levels and functionality of Pacific Islanders’ hybrid humoral immune response to BNT162b2 vaccination and delta/omicron infection: A cohort study in New Caledonia
Source: PLoS Med. 2024 Sep 26;21(9):e1004397. doi: 10.1371/journal.pmed.1004397 (PMC11466435; doi:10.1371/journal.pmed.1004397)
Supplement: S12 Table — (DOCX) [file pmed.1004397.s015.docx]

**S12 Table. Description of participants sampled at 6 months after the third dose of immunization**

|  | **Total**  **(N=488)** | **Melanesian**  **(N=119)** | **European**  **(N=166)** | **Polynesian**  **(N=68)** | **Other**  **(N=135)** | ***p* value** |
| --- | --- | --- | --- | --- | --- | --- |
| **Women, n (%)** | 279 (57.2) | 73 (61.3) | 88 (53.0) | 47 (69.1) | 71 (52.6) | 0.068* |
| **Age (years)**  **Median (IQR)**  **Range** | 54 (40-66)  19-96 | 59 (43-68)  20-88 | 55 (42-68)  20-96 | 46 (39-58)  19-90 | 49 (36-62)  19-92 | <0.001** |
| **Previous infection, n (%)** | 306 (62.7) | 91 (76.5) | 68 (41.0) | 51 (75.0) | 96 (71.1) | <0.001* |
| **Comorbidities, n (%)** | 250 (51.2) | 67 (56.3) | 89 (53.6) | 33 (48.5) | 61 (45.2) | 0.29* |
| **BMI (kg/m²)**  **Median (IQR)**  **Range** | 27.3 (23.4-31.9)  15.8-58.6 | 30.4 (27.1-34.4)  19.3-58.6 | 24.4 (21.5-27.7)  15.8-49.1 | 31.2 (25.7-35.2)  18.9-55.4 | 27.1 (23.0-31.5)  17.0-49.0 | <0.001** |
| **Level of anti-S IgG**  **Median (IQR)**  **Range** | 4.72 (3.32-5.74)  0.90-7.91 | 4.72 (3.57-5.84)  1.00-7.12 | 4.39 (2.86-5.69)  1.23-6.78 | 4.84 (3.78-5.51)  1.04-7.21 | 5.07 (3.68-5.85)  0.90-7.91 | 0.026** |
| **Omicron neutralization >90%, n (%)** | 362 (74.0) | 94 (79.0) | 102 (61.4) | 57 (83.8) | 109 (80.7) | <0.001* |

*IQR: inter quartile range; BMI: body mass index.*

**Chi-2 test; **Kruskal-Wallis test.*
