# Supplementary material for: Levels and functionality of Pacific Islanders’ hybrid humoral immune response to BNT162b2 vaccination and delta/omicron infection: A cohort study in New Caledonia
Source: PLoS Med. 2024 Sep 26;21(9):e1004397. doi: 10.1371/journal.pmed.1004397 (PMC11466435; doi:10.1371/journal.pmed.1004397)
Supplement: S13 Table — (DOCX) [file pmed.1004397.s016.docx]

**S13 Table. Factors associated with the levels of anti-S IgG six months after the third dose (linear regression)**

|  | **N=488** | **Crude effect**  **(95% CI)** | ***p* value** | **Adjusted effect (95% CI)**  **All variables** | ***p* value** |
| --- | --- | --- | --- | --- | --- |
| **Infected**  **No**  **Yes** | 182 (37.3)  306 (62.7) | ***Reference***  **+1.16 (0.91, 1.41)** | **<0.001** | ***Reference***  **+1.24 (0.95, 1.52)** | **<0.001** |
| **Gender**  **Male**  **Female** | 209 (42.8)  279 (57.2) | +0.02 (-0.25, 0.28)  *Reference* | 0.90 | +0.03 (-0.22, 0.28)  *Reference* | 0.80 |
| **Age (years)**  **18-39**  **40-64**  **≥65** | 121 (24.8)  224 (45.9)  143 (29.3) | *Reference*  -0.18 (-0.51, 0.14)  -0.16 (-0.51, 0.20) | 0.52 | *Reference*  -0.08 (-0.39, 0.23)  -0.12 (-0.50, 0.26) | 0.81 |
| **Comorbidities**  **No**  **Yes** | 238 (48.8)  250 (51.2) | *Reference*  -0.09 (-0.35, 0.17) | 0.50 | *Reference*  0.02 (-0.25, 0.29) | 0.90 |
| **BMI**  **Underweight**  **Normal**  **Overweight**  **Obese** | 17 (3.6)  155 (31.8)  138 (28.3)  177 (36.3) | -0.47 (-1.21, 0.26)  *Reference*  +0.28 (-0.06, 0.62)  +0.23 (-0.08, 0.55) | 0.094 | -0.41 (-1.11, 0.28)  *Reference*  +0.20 (-0.12, 0.52)  +0.12 (-0.21, 0.45) | 0.31 |
| **Community**  **European**  **Melanesian**  **Polynesian**  **Other** | 166 (34.0)  119 (24.4)  68 (13.9)  135 (27.7) | ***Reference***  **+0.36 (0.01, 0.70)**  +0.26 (-0.15, 0.68)  **+0.53 (0.19, 0.86)** | **0.016** | *Reference*  -0.09 (-0.45, 0.28)  -0.17 (-0.59, 0.25)  +0.19 (-0.14, 0.52) | 0.28 |
| **Study period**  **March-July**  **August-September** | 224 (45.9)  264 (54.1) | *Reference*  +0.21 (-0.05, 0.47) | 0.11 | *Reference*  -0.28 (-0.55, 0.00) | 0.052 |

*CI: confidence interval; BMI: body mass index.*

*BMI classes: Underweight = BMI<18.5 kg/m², Normal weight = BMI є [18.5, 25[ kg/m², Overweight = BMI є [25, 30[ kg/m², Obese = BMI ≥30 kg/m².*

*Following the backward stepwise procedure, no combination of variables remained significantly associated with the levels of anti-S IgG.*
