# Supplementary material for: Levels and functionality of Pacific Islanders’ hybrid humoral immune response to BNT162b2 vaccination and delta/omicron infection: A cohort study in New Caledonia
Source: PLoS Med. 2024 Sep 26;21(9):e1004397. doi: 10.1371/journal.pmed.1004397 (PMC11466435; doi:10.1371/journal.pmed.1004397)
Supplement: S14 Table — (DOCX) [file pmed.1004397.s017.docx]

**S14 Table. Factors associated with the ability to neutralize Omicron BA.1 and/or BA.4-5 six months after the third dose (logistic regression)**

|  | **N=488** | **NeutralisationN (%)** | **Crude OR**  **(95% CI)** | ***p* value** | **Adjusted OR**  **(95% CI)**  **All variables** | ***p* value** | **Adjusted OR**  **(95% CI)**  **Backward stepwise** | ***p* value** |
| --- | --- | --- | --- | --- | --- | --- | --- | --- |
| **Infected**  **No**  **Yes** | 182 (37.3)  306 (62.7) | 97 (53.3)  265 (86.6) | **1**  **5.66 (3.67, 8.86)** | **<0.001** | **1**  **3.07 (1.82, 5.24)** | **<0.001** | **1**  **4.04 (2.54, 6.50)** | **<0.001** |
| **Level of anti-S IgG**  **<5.737 AU**  **≥ 5.737 AU** | 366 (75.0)  122 (25.0) | 241 (65.8)  121 (99.2) | **1**  **62.76 (13.78, 1111.15)** | **<0.001** | **1**  **56.50 (11.90, 1013.27)** | **<0.001** | **1**  **51.64 (11.00, 922.94)** | **<0.001** |
| **Gender**  **Male**  **Female** | 209 (42.8)  279 (57.2) | 145 (69.4)  217 (77.8) | **0.65 (0.43, 0.97)**  **1** | **0.037** | **0.59 (0.36, 0.97)**  **1** | **0.038** | **0.61 (0.38, 0.97)**  **1** | **0.039** |
| **Age (years)**  **18-39**  **40-64**  **≥65** | 121 (24.8)  224 (45.9)  143 (29.3) | 105 (86.8)  161 (71.9)  96 (67.1) | **1**  **0.39 (0.21, 0.70)**  **0.31 (0.16, 0.57)** | **0.0012** | **1**  **0.46 (0.23, 0.90)**  **0.33 (0.15, 0.72)** | **0.022** | **1**  **0.48 (0.24, 0.90)**  **0.29 (0.14, 0.58)** | **0.003** |
| **Comorbidities**  **No**  **Yes** | 238 (48.8)  250 (51.2) | 184 (77.3)  178 (71.2) | 1  0.73 (0.48, 1.09) | 0.12 | 1  0.95 (0.56, 1.61) | 0.80 |  |  |
| **BMI**  **Underweight**  **Normalweight**  **Overweight**  **Obese** | 17 (3.6)  155 (31.8)  138 (28.3)  177 (36.3) | 10 (58.8)  103 (66.5)  107 (77.5)  142 (80.2) | **0.72 (0.26, 2.09)**  **1**  **1.74 (1.04, 2.96)**  **2.05 (1.25, 3.39)** | **0.012** | 0.63 (0.18, 2.18)  1  1.51 (0.80, 2.87)  1.53 (0.80, 2.94) | 0.35 |  |  |
| **Community**  **European**  **Melanesian**  **Polynesian**  **Other** | 166 (34.0)  119 (24.4)  68 (13.9)  135 (27.7) | 102 (61.4)  94 (79.0)  57 (83.8)  109 (80.7) | **1**  **2.36 (1.39, 4.10)**  **3.25 (1.64, 6.96)**  **2.63 (1.56, 4.53)** | **<0.001** | 1  1.24 (0.61, 2.52)  2.03 (0.89, 4.82)  1.44 (0.76, 2.73) | 0.37 |  |  |
| **Study period**  **March-July**  **August-September** | 224  264 | 143 (63.8)  219 (83.0) | **1**  **2.69 (1.78, 4.13)** | **<0.001** | 1  1.44 (0.84, 2.46) | 0.20 |  |  |

*CI: confidence interval; BMI: body mass index, OR: Odds Ratio.*

*BMI classes: Underweight = BMI<18.5 kg/m², Normal weight = BMI є [18.5, 25[ kg/m², Overweight = BMI є [25, 30[ kg/m², Obese = BMI ≥30 kg/m².*
