# Supplementary material for: Levels and functionality of Pacific Islanders’ hybrid humoral immune response to BNT162b2 vaccination and delta/omicron infection: A cohort study in New Caledonia
Source: PLoS Med. 2024 Sep 26;21(9):e1004397. doi: 10.1371/journal.pmed.1004397 (PMC11466435; doi:10.1371/journal.pmed.1004397)
Supplement: S1 Protocol — (PDF) [file pmed.1004397.s019.pdf]

PROTOCOL SYNOPSIS

1. Full title	Evaluation of the Post-vaccination Immune Response to COVID-19 in the New Caledonian Population
2. Acronym / short title	Covcal
3. Study design	Interventionnal research RIPH 1 (arrêté CSP du 17 mai 2021)
4. Principal Investigator	Dr Marc JOUAN (IPNC)
5. Investigators	Dr Valérie ALBERT-DUNAIS (CHS), Dr Georges MEDEVIELLE (DPASS-Sud), Dr Christophe ASSIE (CHT)
6. Scientific managers	Dr Myrielle DUPONT-ROUZEYROL (IPNC)/ Dr Catherine INIZAN (IPNC)
7. Study rational	The COVID-19 pandemic caused by SARS-CoV-2 since December 2019 has caused more than 210 million cases worldwide as of September 1, 2021. New Caledonia (NC) is an ultramarine French territory in the South Pacific that has been relatively spared from this pandemic until now, thanks to the implementation of a sanitary airlock: unvaccinated travelers were subject to a strict four-week period in a hotel establishment on arrival in the territory, with a PCR test carried out at the end of the four-week period. Similarly, vaccinated travellers were initially subjected to a strict seven-day period in hotels on arrival in the country, with a PCR test at the end of the seven-day period. Until recently, New Caledonia therefore benefited from a "covid-free" situation. However, on September 6, 2021, 3 cases of COVID-19 were detected in the population, and since then more than 12,000 cases have been detected, leading to more than 280 deaths in a population of 280,000. The local vaccination campaign began on 20/01/2021 with the exclusive use of Pfizer's COMIRNATY mRNA vaccine. Priority targets for vaccination were first and foremost "front-line" personnel (in potential contact with travelers entering the country), as well as those most at risk of developing a severe form of COVID-19: people aged over 75 or with certain co-morbidities (diabetes, hypertension, obesity, cancer, kidney failure, severe asthma, etc.). Vaccination is now offered to anyone over the age of 12. To date, some 76% of the target population has received the two doses of vaccine. The third dose, initially intended for vulnerable people, is now offered to all adults from 3 months after the second dose. The vaccination schedule in New Caledonia is standardized: the second dose is administered 3 to 12 weeks after the first dose, and the third dose 3 to 7 months after the second dose in most cases. With the lifting of compulsory vaccination and the gradual abandonment of the health pass, the pace of vaccinations has recently slowed in New Caledonia. Since submission of the first protocol amendment, 125 people have been included in the study after the 2nd dose, and over 328 people after the 3rd dose. As a result of the slowdown in vaccination, inclusions in the study have also fallen sharply over the past 2 weeks.<br><br>Clinical trials of COVID-19 vaccines, and particularly those of mRNA vaccines, have attempted to preserve ethnic diversity within their samples. Efficacy studies showed no significant difference in the efficacy of Pfizer COMIRNATY in white, black American or Hispanic populations. The response of Oceanian populations of non-European non-Asian origin (ONENA) to Pfizer COMIRNATY vaccination has not yet been specifically studied. According to the 2019 census of New Caledonia, 41.2% of the population identified themselves as Kanak (Melanesian), 24% as European, 8.3% as Wallisian-Futunian (Polynesian), 11% as mixed race and 8% as belonging to other communities including Tahitian (Polynesian), Indonesian, Ni-Vanuatu (Melanesian) and Vietnamese (8). Some recent data are in favor of significant variability in pathogen susceptibility among Oceanian populations, stemming from a genetic inheritance from Neanderthal

	man and his cousin Denisova man. In a context of vaccine hesitancy, it is therefore important to ensure that the immune response of the New Caledonian population (of Melanesian, Polynesian, European or other community origin) to vaccination against COVID-19 is similar to that of populations studied in major clinical trials. It is also important to assess the effect of age on the immune response of the New Caledonian population. This study will enable the health authorities to orient or adapt the vaccination strategy, with possible booster doses. The ethnic diversity of the New Caledonian population makes it an ideal observatory of the immune response of New Caledonian populations to the Pfizer COMIRNATY vaccine.
8. Primary objective	To evaluate and compare, in a standardized way, the humoral immune response to COVID-19 vaccination at 1, 6 and 9 months after the third dose of Pfizer COMIRNATY vaccine in the New Caledonian population with populations of Melanesian, Polynesian, European origin or belonging to other communities.
9. Secondary objectives	Longitudinally monitor and compare the humoral immune response to COVID-19 vaccination at 1 and 3 months after the second dose and at 1, 6 and 9 months after the third dose of Pfizer COMIRNATY vaccine in the New Caledonian population.<br>To study the functionality (neutralizing capacity) of antibodies secreted in response to COVID-19 vaccination at 1 and 3 months after the second dose and at 1, 6 and 9 months after the third dose of Pfizer COMIRNATY vaccine in the New Caledonian population.<br>To evaluate and compare, in a standardized way, the humoral immune response to COVID-19 vaccination at 1, 6 and 9 months after the second dose of Pfizer COMIRNATY vaccine in subjects aged 70 and over and a control group (20 to 59 years) in the New Caledonian population.
10. Primary evaluation criterion	ELISA measurement of the presence of antibodies (humoral immunity) at 1, 6 and 9 months after the third dose in vaccinated individuals from the New Caledonian population of Polynesian, Melanesian, European or other community origin:<br>- IgG anti-Spike antibodies (quantitative ELISA)<br>- Anti-nucleoprotein IgG antibodies (measure of natural exposure to the virus)<br>- IgG titer and percentage of participants with a positive (criterion A) or strong positive (criterion B) ELISA based on thresholds established in the literature.
11. Secondary evaluation criteria	Measurement by ELISA of the presence of antibodies (humoral immunity) at 1 and 3 months after the second dose and at 1, 6 and 9 months after the third dose of Pfizer COMIRNATY vaccine in vaccinated individuals from the New Caledonian population of Polynesian, Melanesian, European or other community origin:<br>- - IgG anti-Spike antibodies (quantitative ELISA)<br>- - Anti-nucleoprotein IgG antibodies (measure of natural exposure to the virus)<br>- - IgG titer and percentage of participants with a positive (criterion A) or strong positive (criterion B) ELISA based on thresholds established in the literature.<br>Serum-neutralization analysis of the neutralizing capacity against the different circulating SARS-CoV-2 variants of antibodies secreted by Caledonian individuals at 1 and 3 months after the second dose, and at 1, 6 and 9 months after the third dose of Pfizer COMIRNATY vaccine.<br>Post-hoc analysis of anti-Spike and anti-Nucleoprotein ELISA results at 1, 6 and 9 months by age stratification of the New Caledonian population (group over 70 years and control group 20 to 59 years)
12. Materials	4 mL dry tube of blood collected at 1, 6 and 9 months after the third dose of Pfizer COMIRNATY vaccine and at 1 and 3 months after the second dose of Pfizer COMIRNATY vaccine for a subsample.
13. Method	Observational equivalence study with prospective data collection.<br>Vaccinated subjects to be included will be identified at vaccination centers in the

	Southern Province at the time of injection of the second or third dose. Vaccinated subjects wishing to know their antibody levels at 6 and 9 months after the third dose are invited to come forward at a distance, 1 to 5 ½ months after their 3rd dose, in order to be included in the study.<br>Information and consent<br>Completion of a questionnaire and collection of 4 mL of blood on a dry tube at 1 and 3 months after the second dose (for a subsample) and at 1, 6 and 9 months after the third dose for the entire cohort.<br>ELISA tests for anti-SARS-CoV-2 antibodies (anti-Spike and anti-Nucleoprotein)<br>Results of serological analyses returned to participants.
14. Study population	Adults of Melanesian, Polynesian, European or other community origin vaccinated with a complete COVID-19 vaccination regimen, resident in New Caledonia
15. Number of subjects	For the overall sample (follow-up after the third dose): 193 individuals of Melanesian origin, 193 individuals of Polynesian origin, 193 individuals of European origin, 193 individuals from other communities.<br>For the sub-sample for longitudinal follow-up after the second and third doses: 138 individuals representative of the community diversity of the New Caledonian population.<br>A total of 910 individuals will be included in the study.
16. Inclusion criteria	- Adult of Melanesian, Polynesian, European or other community origin<br>- For the overall sample (follow-up after the 3rd dose): individual vaccinated with 3 doses of Pfizer COMIRNATY vaccine, with the second dose administered between 3 and 12 weeks after the first, and the third dose administered 3 to 6 months after the second dose.<br>- For the sub-sample followed longitudinally (follow-up after 2nd and 3rd dose): Individual vaccinated with 2 doses of Pfizer COMIRNATY vaccine, administered between 3 and 12 weeks apart<br>- Individual planning to be present on the territory and available on both sampling dates after each dose)<br>- Informed individual capable of giving free and informed consent alone, and having consented to participation in the study
17. Non-inclusion criteria	The following will be excluded from the survey<br>- For the overall sample (follow-up after the 3rd dose): people who received the third dose before 3 months or after 6 months after the second dose.<br>- For the sub-sample followed longitudinally (follow-up after 2nd and 3rd dose): people who received the second dose of vaccine more than 12 weeks after the first dose.<br>- People vaccinated with a COVID-19 vaccine other than Pfizer COMIRNATY<br>- Women claiming to be pregnant or breastfeeding (in whom the immune response could be altered)<br>- Immunocompromised individuals (immunosuppressive or immunomodulating therapy, HIV-positive, splenectomy, long-term corticosteroid therapy, autoimmune diseases such as rheumatoid arthritis, lupus, spondylitis, etc.).<br>- People who are not intellectually capable of answering the questionnaire<br>- Persons under guardianship, curatorship or any other legal incapacity<br>- Individuals with documented Covid-19 infection (known positive SARS-CoV-2 serology prior to inclusion, positive PCR or antigen test).<br>For safety reasons, participants in this research must agree not to enter a vaccine clinical trial during the entire inclusion and follow-up period.

18. Study Calendar	▪ Provisional start date for inclusions : December 2021<br>▪ Anticipated duration of inclusion: 12 months<br>▪ Duration of subject participation: 9 months for total sample, 12 months for sub-sample<br>▪ Anticipated study duration: 3 years
19. Number of centres	Heath centers of Southern Provinve in New Caledonia (vaccinodromes, medical and social centers, centres médico-sociaux, Centre Hospitalier Territorial et Centre Hospitalier Spécialisé, …).
20. Expected results and possible implications	Monitoring the humoral response of subjects of Melanesian, Polynesian, European and other community origin to vaccination against COVID-19<br>Study of the humoral response to COVID-19 vaccination of populations not previously studied in efficacy studies<br>Indicators for possible adaptation of the vaccination protocol and booster doses to the particularities and age of Pacific populations
21. Collaborators	Institut Pasteur de Nouvelle-Calédonie<br>Centre Hospitalier Spécialisé Albert Bousquet<br>Centre Hospitalier Territorial<br>Direction Provinciale de l'Action Sanitaire et Sociale Sud<br>Agence Sanitaire et Sociale de Nouvelle-Calédonie
