# Supplementary material for: Levels and functionality of Pacific Islanders’ hybrid humoral immune response to BNT162b2 vaccination and delta/omicron infection: A cohort study in New Caledonia
Source: PLoS Med. 2024 Sep 26;21(9):e1004397. doi: 10.1371/journal.pmed.1004397 (PMC11466435; doi:10.1371/journal.pmed.1004397)
Supplement: Attachment — Submitted filename: inizan.pdf [file pmed.1004397.s021.pdf]

This paper discusses immune response to vaccination and infection with SARS–CoV–2 in various communities.

I was asked for a methodological report and I interpret that to include all aspects of the design and conduct of the study. The authors have made the decision to depart from the usual order of sections in a scientific report except in the abstract. I think there is usually a good reason to follow the conventional order so I have arranged my comments accordingly.

Points of detail

Summary and introduction

Page 1 I would put New Caledonia in the title. Although there is a move to use vernacular geographical names in English I do not think we are ready for Nouvelle Calédonie yet sadly.

Page 3 Since the point of the paper is differences between ethnic groups I think we need more detail here about those differences or their absence and less about other covariates.

Page 6 Why use both White and European here? Do the authors intend us to see them as different? The phrase 'of European descent' is often useful.

Methods

Page 14 I am afraid this section leaves me ignorant of many important details and confused about the authors' intentions. The study is registered as a trial but there does not seem to be an intervention (which is not compulsory of course). How were people recruited into the study? Is this a consecutive sample of attenders at a routine clinic or were they invited to take part in a research project?-

Page 15 We read 'Not all participants enrolled in the study participated in the follow–up.' Why? Loss to follow–up is important especially in an equivalence trial and needs to be explained somewhere.

Page 17 Why categorise age? Categorising an essentially continuous variable wastes information (Altman and Royston, 2006; Royston et al., 2006) and leads to models which are often implausible as they predict the effect remaining flat within categories and then jumping to a new value at the category boundary. For presentation it may be permissible but for modelling it is undesirable.

Page 17 Selecting a subset of variables in a way driven by the data leads to a model which is unlikely to replicate (Babyak, 2004). Screening variables to enter the model has been criticised too (Sun et al., 1996) What was the scientific question which made selection necessary?

Results

Page 8 According to the methods (page 14) the primary outcome was the fraction exhibiting an anti–S IgG response. I have searched here and I do not find this reported. We do get an analysis of levels but that is not the same.

Page 8 The ROC analysis does not seem to be described in the methods. It is not clear to me what it is telling me.

In four pages of results we only seem to get six sentences about the differences between communities and those are rather bald. Is there some reason for this emphasis or lack of it?

Discussion

Page 13 The term metis was not familiar to me. Investigating in Wikipedia the specific entries for it seem to suggest it is used differently in English, where it seems to refer primarily to quite specific communities in the US and Canada, and in French, where métis is equivalent to the English term mixed race or one of its variants. Wikipedia might be wrong of course but it might be better to use an alternative term.

Page 13 The authors state as a limitation '... finally the sample size was not reached, hindering to ascertain response equivalence between communities.' No, the reverse is the case. Under–recruitment in an equivalence trial biases towards finding equivalence (Jones et al., 1996).

Points of more substance

Representativeness

I have already remarked that we do not know how people were recruited into the study. This is made more salient since the participants do not appear with the population frequencies given on page 6. This is rather obvious but for the record a formal test gives a $\chi^2_3 = 63.9$ with associated probability way beyond any conventional α level ($p = 8.5 \times 10^{-14}$). Is this because there was some form of stratified sampling?

Width of confidence intervals

The authors had a target of 141 per group (page 14). The actual numbers are much lower and I calculate that, all else being equal, the width of the confidence intervals has been inflated by about 1.35 times.

	est	newll	newul
European (reference category)	0.00		
Melanesian	0.02	-0.19	0.23
Polynesian	0.24	0.03	0.45
Other	0.20	0.03	0.37

Table 1: Example using deflated standard errors

In my table 1 I show what would happen if the standard errors had been deflated by a factor of 1.35. As can be seen this alters the conclusions. I am not saying that if the design sample size had been achieved the confidence intervas would have been as my table shows but I do think that we need much more caution here about any attempt to claim that the various communities had the same response.

Is the other category relevant?

The other category of ethnicity is presumably a mixture of people who refused to say, people who identify as another category, and people of mixed race. It is hard to see them being relevant to the primary issue here, whether Pacific Islanders have a different response to people of European descent. I suggest removing them and only including them in a sensitivity analysis.

Summary

I have raised a number of points which I think need clarifying and where details are currently in the authors' heads rather than on the paper. It is unlikely that many similar data sets will be available and so I would not regard the failure to achieve the planned sample size as a fatal flaw but I do think it needs to be interpreted properly.

Michael Dewey

References

D G Altman and P Royston. The cost of dichotomising continuous variables. *British Medical Journal*, 332:1080, 2006.

M A Babyak. What you see may not be what you get: a brief, nontechnical introduction to overfitting in regression–type models. *Psychosomatic Medicine*, 66:411–421, 2004.

B Jones, P Jarvis, J A Lewis, and A F Ebbutt. Trials to assess equivalence: the importance of rigorous methods. *British Medical Journal*, 313:36–39, 1996.

P Royston, D G Altman, and W Sauerbrei. Dichotomizing continuous predictors in multiple regression: a bad idea. *Statistics in Medicine*, 25:127–141, 2006.

G-W Sun, T L Shook, and G L Kay. Inappropriate use of bivariable analysis to screen risk factors for use in multivariable analysis. *Journal of Clinical Epidemiology*, 49:907–916, 1996.
