# Supplementary material for: Levels and functionality of Pacific Islanders’ hybrid humoral immune response to BNT162b2 vaccination and delta/omicron infection: A cohort study in New Caledonia
Source: PLoS Med. 2024 Sep 26;21(9):e1004397. doi: 10.1371/journal.pmed.1004397 (PMC11466435; doi:10.1371/journal.pmed.1004397)
Supplement: Attachment — Submitted filename: inizan.pdf [file pmed.1004397.s023.pdf]

This paper discusses immune response to vaccination and infection with SARS–CoV–2 in various communities.

I was asked for a methodological report and I interpret that to include all aspects of the design and conduct of the study.

Points of detail

I said:

> *We read 'Not all participants enrolled in the study participated in the follow–up.' Why? Loss to follow–up is important especially in an equivalence trial and needs to be explained somewhere.*

Figure 1 does contain some information as stated in the rebuttal but it does not specify the reasons for people not appearing at the various stages. How many refused to be recruited? How many became untraceable?

I said:

> *Selecting a subset of variables in a way driven by the data leads to a model which is unlikely to replicate (Babyak, 2004). Screening variables to enter the model has been criticised too (Sun et al., 1996) What was the scientific question which made selection necessary?*

I did not find the authors' rebuttal not very convincing here. Just because a variable does not reach some arbitrary level of statistical significance in a univariate analysis does not mean it will or will not contribute in the multivariable one. If the univariate analyses could tell us what the multivariable analysis was going to say we would not have needed to invent multiple regression. Why not present a full model with all covariates which might be important?

I said:

> *The ROC analysis does not seem to be described in the methods. It is not clear to me what it is telling me.*

What motivated the choice of cut–off for sensitivity and specificity? On the face of it missing true cases is more important that picking false positives so the present choice needs justifying.

I said:

> *I have already remarked that we do not know how people were*

> *recruited into the study. This is made more salient since the participants do not appear with the population frequencies given on page 6. This is rather obvious but for the record a formal test gives a* $\chi^2_3 = 63.9$ *with associated probability way beyond any conventional* α *level (*$p = 8.5 \times 10^{-14}$*). Is this because there was some form of stratified sampling?*

In their rebuttal the authors mention stratified sampling. How exactly was this managed? When people presented for their vaccination were their details taken and then a decision made as to eligibility? Or did their medical records already have information on their self–reported ethnic group? Did the central office monitor recruitment and then issue an order to stop recruiting from that ethnicity when 141 had been recruited? Note that if this means that the Polynesian group were still being recruited when all the other ethnicities had stopped this is not best practice if there might be secular effects.

Conclusion

As I remarked in my original review this is a data–set which is unlikely to be repeated and if the clinical reviewers feel it is an important study then we can take a more relaxed attitude to any problems I feel I have identified. Nonetheless I think for transparency we do need more details, as I have outlined.

Michael Dewey

References

M A Babyak. What you see may not be what you get: a brief, nontechnical introduction to overfitting in regression–type models. *Psychosomatic Medicine*, 66:411–421, 2004.

G-W Sun, T L Shook, and G L Kay. Inappropriate use of bivariable analysis to screen risk factors for use in multivariable analysis. *Journal of Clinical Epidemiology*, 49:907–916, 1996.
