# Supplementary material for: Levels and functionality of Pacific Islanders’ hybrid humoral immune response to BNT162b2 vaccination and delta/omicron infection: A cohort study in New Caledonia
Source: PLoS Med. 2024 Sep 26;21(9):e1004397. doi: 10.1371/journal.pmed.1004397 (PMC11466435; doi:10.1371/journal.pmed.1004397)
Supplement: Attachment — Submitted filename: COVCAL Response to reviewers 20240626.docx [file pmed.1004397.s022.docx]

Dr Catherine Inizan

Institut Pasteur in New Caledonia

11, ave Paul Doumer

BP 61, 98845 NOUMEA Cedex

NOUVELLE-CALEDONIE

Tel +687 272666

cinizan@pasteur.nc

Nouméa, the 26^th^ of June, 2024

Dear Reviewers,

Please find below a point-by-point response to your comments on our manuscript initially entitled “**Hybrid humoral immune response of Pacific Islanders to BNT162b2 vaccination and Delta/Omicron infection: a cohort study**" (PMEDICINE-D-24-01093R1).

We thank you for reviewing our manuscript. You have raised a number of very useful points that needed to be taken care of in order to improve the manuscript. We propose here a point-by-point response to your comments, describing any additional analyses that were carried out, and propose a revised version of the manuscript. Given the number of important points raised by the editor and the four reviewers, substantial changes have been made to the manuscript, and especially to the discussion section. We believe the manuscript in its current version is enhanced by those changes.

We have ensured that all changes to the manuscript are indicated in the text by using track changes. We also want to draw your attention on the fact that we modified the title of the manuscript to “**Levels and functionality of Pacific Islanders’ hybrid humoral immune response to BNT162b2 vaccination and Delta/Omicron infection: a cohort study in New Caledonia**” and have appended an “Author summary” to this revised version of the manuscript. In addition, we provide the de-identified datasets used in the analyses presented in the manuscript, the STROBE checklist as well as the summary of the study protocol as supplementary files.

We hope this revised version will meet your recommendations and suggestions for improvement.

Sincerely yours,

Dr Catherine Inizan, on behalf of all authors.

EDITORIAL COMMENTS:
The editors agree that the authors should be commended for performing valuable work with a population that has been under-represented. We are very grateful that you chose to submit your research for evaluation to PLOS Medicine. However, we do agree with the points raised by reviewers 1, 3 and 4, and ask that the authors respond to these in full.

1)      Data Availability:
The Data Availability Statement (DAS) requires revision. Given the data are not freely available, please describe briefly the ethical, legal, or contractual restriction that prevents you from sharing it. Please also include an appropriate contact (web or email address) for inquiries (but note, this cannot be a study author). Please see the following page for further information and guidance:
[https://urldefense.com/v3/__https://journals.plos.org/plosmedicine/s/data-availability*loc-unacceptable-data-access-restrictions__;Iw!!JFdNOqOXpB6UZW0!s6fZvbpxYxK8iwaVielESGQ5T0EQDG8299UMP00rIIZQz0G4wYrxYYxG9bOOUOhdVdJUtlkfmvb7mJAoxA$](https://urldefense.com/v3/__https:/journals.plos.org/plosmedicine/s/data-availability*loc-unacceptable-data-access-restrictions__;Iw!!JFdNOqOXpB6UZW0!s6fZvbpxYxK8iwaVielESGQ5T0EQDG8299UMP00rIIZQz0G4wYrxYYxG9bOOUOhdVdJUtlkfmvb7mJAoxA$)

Discussion with the Data Protection Officer from the Institut Pasteur, which sponsored the current study, confirmed that the datasets used to generate the results reported in the current manuscript can be shared, provided that they are limited to the data used in the analyses shown in the manuscript. We therefore share the deidentified datasets as a supplementary .xls file.

2)      Reporting guidance:
Please report your study according to the relevant guidance which can be found here [https://urldefense.com/v3/__https://www.equator-network.org/reporting-guidelines/__;!!JFdNOqOXpB6UZW0!s6fZvbpxYxK8iwaVielESGQ5T0EQDG8299UMP00rIIZQz0G4wYrxYYxG9bOOUOhdVdJUtlkfmvaQt5luLQ$](https://urldefense.com/v3/__https:/www.equator-network.org/reporting-guidelines/__;!!JFdNOqOXpB6UZW0!s6fZvbpxYxK8iwaVielESGQ5T0EQDG8299UMP00rIIZQz0G4wYrxYYxG9bOOUOhdVdJUtlkfmvaQt5luLQ$)

We identified the STROBE reporting guidelines as the more appropriate to report the results of our cohort study. We added some elements to the text of the manuscript to comply with these reporting guidelines:

- Line 150-151 page 7
- Line 158 page 8
- Line 160-161 page 8
- Line 164-166 page 8
- Line 175-176 page 8
- Line 179-181 page 9
- Line 275-276 page 12
- Line 280-282 page 12-13
- Line 294 page 13
- Line 295-298 page 13
- Line 546 page 23

We also filled the STROBE checklist for cohort studies and provide it as supplementary material. We mentioned the compliance with the STORBE reporting guidelines and the addition of the checklist line 156-158 page 8.

Finally, we modified the title of Figure 1: “**STROBE (****STrengthening the Reporting of OBservational studies in Epidemiology**) **diagrams of the second- and third-dose cohorts.”**

3)      Statistical reporting:
(Note that not all of the instructions below may be applicable to your work.)

Please quantify the main results with 95% CIs and p values.

We provide 95% CI and *p* values for all results reported in the tables and supplementary tables, as well as for the main results reported in the manuscript’s main text.
When reporting p values please report as <0.001 and where higher as p=0.002, for example. When reporting 95% CIs please separate upper and lower bounds with commas instead of hyphens as the latter can be confused with reporting of negative values.

We report *p* values as recommended by the editor. We changed the separator for the upper and lower bounds of 95% CIs from semi-colons or hyphens to commas.

Please include the actual amounts and/or absolute risk(s) of relevant outcomes (including NNT or NNH where appropriate), not just relative risks or correlation coefficients. (example for absolute risks: PMID: 28399126).

This recommendation is not applicable to our study.

Please include any important dependent variables that are adjusted for in the analyses.

We adjusted the univariate linear regression conducted on anti-S IgG levels on timepoint and infection status. We specified it in the legend of Table 1. We added the following sentence to the manuscript page 14 line 326-327: “Given the high dependence of anti-S IgG levels on timepoint of analysis and infection status of the participants, the univariate analysis was adjusted for timepoint and infection status.”

4)      Prespecified analysis plan/study protocol:
I note that you have included a protocol in the supporting information. Can you provide an English translation of this? Please also state that you had a prospective study protocol early in the Methods section. Also, note that any changes in the analysis-- including those made in response to peer review comments-- should be identified as such in the Methods section of the paper, with rationale.
The protocol provided in the supporting information is the protocol submitted to the French ethics committee (Comité de Protection des Personnes) and to the Consultative Ethics Committee of New Caledonia. We provided it for the editor’s information. Instead, we suggest providing the study protocol submitted on Clinical Trials, for the reader’s understanding. The protocol submitted in Clinical Trials is now provided as a new Supporting material.
We also specified line 150-151page 7 that “A prospective study protocol was set up ahead of study implementation.”

Additional analyses implemented in response to reviewers’ recommendations have been described in the methods section Line 273-274 page 12 “The effect of self-declared ethnicity on anti-N IgG levels was investigated using linear regression models.” and Line 281-282 page 12-13 “Sensitivity analyses not considering participants self-declaring as belonging to other communities were performed at one-month post immunization.”

5)      Abstract layout:
Please structure your abstract using the PLOS Medicine headings (Background, Methods and Findings, Conclusions).

The heading of the “Results” section in the abstract has been changed for “Findings”.

6)      Author summary:
At this stage, we ask that you include a short, non-technical Author Summary of your research to make findings accessible to a wide audience that includes both scientists and non-scientists. The authors summary should consist of 2-3 succinct bullet points under each of the following headings:
• Why Was This Study Done? Authors should reflect on what was known about the topic before the research was published and why the research was needed.
• What Did the Researchers Do and Find? Authors should briefly describe the study design that was used and the study’s major findings. Do include the headline numbers from the study, such as the sample size and key findings.   
• What Do These Findings Mean? Authors should reflect on the new knowledge generated by the research and the implications for practice, research, policy, or public health. Authors should also consider how the interpretation of the study’s findings may be affected by the study limitations. In the final bullet point of ‘What Do These Findings Mean?’, please describe the main limitations of the study in non-technical language.
The Author Summary should immediately follow the Abstract in your revised manuscript. This text is subject to editorial change and should be distinct from the scientific abstract. Please see our author guidelines for more information: [https://urldefense.com/v3/__https://journals.plos.org/plosmedicine/s/revising-your-manuscript*loc-author-summary__;Iw!!JFdNOqOXpB6UZW0!s6fZvbpxYxK8iwaVielESGQ5T0EQDG8299UMP00rIIZQz0G4wYrxYYxG9bOOUOhdVdJUtlkfmvbFSZEGwg$](https://urldefense.com/v3/__https:/journals.plos.org/plosmedicine/s/revising-your-manuscript*loc-author-summary__;Iw!!JFdNOqOXpB6UZW0!s6fZvbpxYxK8iwaVielESGQ5T0EQDG8299UMP00rIIZQz0G4wYrxYYxG9bOOUOhdVdJUtlkfmvbFSZEGwg$)

We added the Author summary page 5.

**Author summary**

Why Was This Study Done?

- Pacific Islanders are at risk for severe COVID-19
- In a few studies, ethnicity had no impact on antibody levels following Pfizer BNT162b2 vaccine in Pacific Islanders; in these studies however, follow-up was reduced and no distinction was made between the different Pacific ethnic communities.
- Characterizing the levels and functionality of Pacific Islanders humoral immune response to Pfizer BNT162b2 vaccine is pivotal to adapt vaccination regimen to regional singularities

What Did the Researchers Do and Find?

- The current study enrolled 585 participants from New Caledonia self-declaring as Melanesians, Polynesians, Europeans or belonging to Other Communities and characterized their antibodies levels and functionality at one and three months post-second dose or one and six months post-third dose of the Pfizer BNT162b2 vaccine
- 61.3% of the sera contained traces of previous SARS-CoV-2 infection; the cohort’s immune response was thus mostly hybrid, resulting from both vaccination and infection
- At all timepoints analyzed, the hybrid humoral immune response remained equivalent across the Melanesian, Polynesian, European and Other communities: anti-Spike IgG levels, capacity to mediate Omicron neutralization and capacity to mediate ADCC were not significantly different across communities (*p* values>0.05).

What Do These Findings Mean?

- The lack of community effect, despite different prevalence of obesity, suggests that SARS-CoV-2 hybrid humoral response does not differ between communities.
- Such robust and prototypical humoral immune response to Pfizer BNT162b2 vaccine and SARS-CoV-2 in Pacific Islanders is of prime importance to foster booster doses roll-out, with the aim to better protect this under-studied population which is vulnerable to severe COVID-19.
- Biological confirmation of ethnicity and a larger sample size in the Polynesian community would enhance the resolution of our findings.

7)      Introduction layout: Please address past research and explain the need for and potential importance of your study.   If there has been a systematic review of the evidence related to your study (or you have conducted one), please refer to and reference that review and indicate whether it supports the need for your study.

Although no formal systematic review was performed, we added to the introduction the results of the search we conducted on Pubmed with a selection of key terms, which evidenced the lack of knowledge on Pacific Islanders’ immune response to COVID-19 vaccination. We added the results of this search in the introduction Line 120-124 page 6. Past research conducted by others on this topic is presented line 124-130 page 6-7. We further developed the knowledge gap filled by our study line 130-133 page 7.

8)      Methods section:
Please move your Methods section so that it appears after your Introduction and before your Results in the main text.

The Methods section has been moved after the Introduction page 7-13.

9)      Discussion layout:
Thank you for starting your Discussion with a short summary of the article's findings. For the result of the Discussion section, please present the following points in this order: what the study adds to existing research and where and why the results may differ from previous research; strengths and limitations of the study; implications and next steps for research, clinical practice, and/or public policy; one-paragraph conclusion.

As per the editor’s recommendations, we have reorganized the discussion and added sentences to comply with the following order:

- Short summary of article’s findings Line 412-415
- What the study adds to existing research Line 416-427
- Where and why the results may differ or be consistent from previous research Line 428-485 (in which we discuss the implications of SARS-CoV-2 infection on the humoral immune response to vaccination, sex-related discrepancies in the humoral immune response, age-related differences in the humoral immune response, and the impact of BMI on the humoral immune response)
- The strengths and limitations Line 486-512
- Implications and next steps for research, clinical practice and/or public policy Line 513-524
- Conclusion Line 525-530

Reviewer #1: This manuscript represents an important addition to the literature by presenting information on vaccine efficacy of an underrepresented population. The report is missing crucial information regarding the protection of this population by antibodies.
We thank the reviewer for her/his positive appraisal of our work. As our study was not designed as a vaccine efficacy trial, vaccine efficacy and correlates of protection cannot be drawn from our results. We nonetheless provide an unprecedented characterization of Pacific Islanders’ antibody response to Pfizer BNT162b2 vaccination.

Minor comments:
- Was the study undertaken in line with the Declaration of Helsinki? If so, please specify
The study was indeed undertaken in line with the Declaration of Helsinki principles. This was specified at the beginning of the Methods section, which is now line 148 page 7. We apologize for making it not visible enough and decided to split in two the first sentence of the Methods section to put more emphasis on this important point: “The current study was conducted in compliance with the Declaration of Helsinki principles. The study was recorded on clinicaltrials.gov (ID: NCT05135585).”

Major comments:
- Were the spike and nucleoprotein-specific responses both IgG and neutralisation measured in this study towards the Ancestral Wuhan virus? Please specify strain in methods section and on figures.

IgG levels against SARS-CoV-2 Spike and Nucleoprotein were measured in LuLISA experiments using recombinant SARS-CoV-2 Spike and Nucleocapsid proteins from the ancestral Wuhan virus.

Neutralization experiments were performed using 4 pseudotyped lentiviral vectors each enveloped with the Spike protein of one of the following variants: Wuhan, Delta, Omicron BA.1 or Omicron BA.4/5.

ADCC experiments were performed using a cell line stably expressing the Spike protein from the ancestral Wuhan strain.

Although we mentioned this in the methods section, we apologize for not making it clear enough for the reader’s understanding. We therefore added the following sentences in the Methods section, Figures and Figure legends:

- With regard to the measurement of IgG levels in LuLISA experiments: Line 205-206 page 10 “Anti-Spike and Nucleoprotein IgG levels were assessed in LuLISA experiments performed using recombinant SARS-CoV-2 Spike and Nucleoprotein proteins from the ancestral Wuhan strain.”

We also added to the legend of Figure 3: page 27 line 583-584: “Concentrations in IgG targeting the Spike protein of SARS-CoV-2 ancestral strain Wuhan in samples collected one month after the second dose from previously infected participants (D2.1 infected, red), one month after the second dose from previously non-infected participants (D2.1 non infected, green), one month after the third dose from previously infected participants (D3.1 infected, blue) and one month after the third dose from previously non-infected participants (D3.1 non infected, purple).” and page 27 line 585-586 “Concentrations in IgG targeting the Spike protein of SARS-CoV-2 ancestral strain Wuhan in samples collected from self-declared Melanesian (dark blue), European (light blue), Polynesian (light green) participants or from participants self-declaring as belonging to other communities (dark green) one month after immunization.”

We modified the labels of the Y axes of Figure 3 accordingly.

- With regard to the neutralization experiments: Line 225-228 page 10 “Capacity of participants’ sera to neutralize either SARS-CoV-2 Wuhan, Delta, Omicron BA.1 or Omicron BA.4/5 variants was assessed in four pseudovirus neutralization assays, each using a different pseudotyped virus enveloped with the Spike protein of one of the following variants: ancestral Wuhan strain; Delta variant, Omicron BA.1 variant or Omicron BA.4/5 variant.”

Regarding neutralization, Figure 3 already specifies that neutralization capacity was measured against SARS CoV-2 variants Wuhan, Delta, Omicron BA.1 and Omicron BA.4/5. We specified that Wuhan is the ancestral strain in the X axis of Figure 3.C

We modified Figure legend page 27 line 592-593 as follows “Heatmap of the neutralization capacity against SARS-CoV-2 ancestral strain Wuhan, and against SARS-CoV-2 variants Delta, Omicron BA.1 and Omicron BA.4/5, one month after the second dose in previously non-infected participants (D2.1 non infected, green), one month after the second dose in previously infected participants (D2.1 infected, red), one month after the third dose in previously non-infected participants (D3.1 non infected, violet) and one month after the third dose in previously infected participants (D3.1 infected, blue).”.

- With regard to ADCC assay: Line 248-249 page 11 “Capacity of participants’ sera to activate CD16 was quantified using 293T cells stably expressing the Spike protein from SARS-CoV-2 ancestral strain Wuhan.”

We added to the title of Table 3 Line 617-618 page 31 “Factors associated with the level of ADCC (CD16 activation in response to SARS-CoV-2 Spike protein from the ancestral strain Wuhan) one month after immunization (Linear regression)**”**

We also added to the title of Supplementary table 11: Line 656-657 page 33 “Factors associated with the variation in the level of ADCC (CD16 activation in response to SARS-CoV-2 Spike protein from the ancestral strain Wuhan) between one and six months after immunization (linear regression)”.

- Include information regarding the demographics of the study population including age and gender in first results paragraph

We thank the reviewer for this suggestion and added the following sentences in the first paragraph of the results section, line 287-289 page 13 “The proportion of women was 55.9% (327/585). In the cohort, median age was 51 years (range 19-96) and median BMI was 27.5 (IQR 23.4, 32.0). The proportion of participants with comorbidities was 49.9% (292/585).”.

- If available include information about thich virus variant wave participants were infected in

Past infection of participants was determined based on the presence of anti-Nucleoprotein antibodies in samples collected at one-, three- or six-months post-immunization. No nasopharyngeal swab was collected during acute SARS-CoV-2 infection in the framework of the current study. It is therefore impossible to formally ascertain which variant the participants were infected by. Due to COVID-19 epidemiology in New Caledonia and the timeline of participants inclusion and sampling (see Supplementary Figure 1), it is however possible to assume that infected participants sampled at D2.1 were likely infected by the Delta variant, and participants sampled at later timepoints were infected either by the Delta or Omicron variants.

We rephrased the sentence related to this information in the Discussion section line 439-443 page 19: “The infecting serotype was not determined in the framework of the current study. However, due to COVID-19 epidemiology in NC (Supplementary figure 1), it is possible to infer that while participants with a history of past infection sampled at D2.1 were likely infected by the Delta variant, and participants with a history of past infection sampled at later timepoints could have been infected either by the Delta or Omicron variants.”

- What was the timing between vaccine dose as this can have an impact on the magnitude of response measured.

Timing between vaccine doses was a strict inclusion criterion: participants received the second dose between 3 and 12 weeks after the first dose, and the third dose between 3 and 6 months after the third dose, as per New Caledonia Health Authorities’ recommendations. We apologize for not mentioning it. We added the following sentence in the methods section Line 164-166 page 8 “Having received the second dose between three and 12 weeks after the first dose, and the third dose between three and six months after the third dose was an inclusion criterion.”

- The authors note that neutralisation levels of Omicron is reduced 6 months after dose 6. What Omicron variant was this too? It is important to include data on the anti-Omicron spike IgG levels. 1. Virus-specific IgG levels will start to wane by 6 months post dose 3. 2. In the literature recognition of Omicron variants is significantly decreased see Hartley et al. 2023 BioRxIV (https://urldefense.com/v3/__https://www.biorxiv.org/content/10.1101/2023.09.15.557929v1__;!!JFdNOqOXpB6UZW0!s6fZvbpxYxK8iwaVielESGQ5T0EQDG8299UMP00rIIZQz0G4wYrxYYxG9bOOUOhdVdJUtlkfmvYoyM92mg$ )

Different from the study cited by the reviewer, our serological assays are not based on the use of recombinant tetramers or RBD from each SARS-CoV-2 variant. It is therefore not possible to determine the levels of Spike IgG levels specifically targeting Omicron in our experimental setting. However, in our study, pseudovirus neutralization assays were performed using four different pseudotyped viruses each enveloped with the Spike protein of either the ancestral Wuhan strain or of the Delta, Omicron BA.1 or Omicron BA.4/5. This allowed us to determine the level of Omicron neutralization, which is both related to anti-Omicron Spike IgG levels and a fair proxy of protection against this variant. This level of Omicron neutralization was measured at one-month post-immunization (Table 2) and we evidenced its waning between one and six months post-third dose (Supplementary tables 6&10). Finally, we assessed its level at six months post-immunization (Supplementary tables 12&14).

In order to clarify the Omicron variant used to determine Omicron neutralization capacity, we rephrased our definition of Omicron neutralization in the methods section, page 11 Line 241-243 “A participant’s serum was considered able to neutralize Omicron variant if a 1/40 dilution of his/her serum exhibited a ≥90% neutralization of either Omicron BA.1 and/or Omicron BA.4/5 pseudoviruses in pseudovirus neutralization assays (ie neutralization titer >40).”

In addition, we would like to thank the reviewer for drawing our attention on the study she/he cites. We added the reference cited by the reviewer to the Discussion section, page 19 line 449-451 “A third dose of BNT162b2 vaccine resulted in Omicron neutralization in almost all participants, independently from a previous infection. This is in line with the acquisition by memory B cells of the capacity to recognize Omicron BA.2 and BA.5 variants after a booster dose of BNT162b2 ^22^.”

- There is no mention that other metrics of immunity should be measured to ensure a more holistic overview of the reponse of this population to vaccination

We thank the reviewer for this suggestion and added to the discussion line 520-524 page 22 “This study focused on the humoral immune response. Singularities of Pacific Islanders’ innate and cellular immune responses elicited by SARS-CoV-2 infection and BNT162b2 vaccination, and their potential contribution to COVID-19 peculiar epidemiology in Pacific Islanders communities remain to be assessed to build a more holistic overview of Pacific Islanders’ response to COVID-19 vaccination.”

Reviewer #2: The manuscript assesses humoral vaccine responses to BNT162b2 vaccination in a cohort of participant from New Caledonia.

I congratulate the authors on a good study with a large hard to collect and complicated set of data. The study is well conducted and findings are robust and of interest and importance to the research community.

However I do not think the findings "provide a substantial advance over existing knowledge".

Only a few of the findings add to excising knowledge and whilst these are important, are not substantial advances.
We thank the reviewer for this positive assessment of our work. Our data are indeed in line with published results characterizing the humoral immune response to Pfizer BNT162b2 vaccine. Such evidence of a robust and efficient humoral immune response in Pacific Islanders, consistent with the literature, is however pivotal knowledge to enforce and provide scientific evidence of the usefulness of vaccination campaigns in a population at risk for severe COVID-19.

Reviewer #3: The manuscript by Inizan et al titled 'Hybrid humoral immune response of Pacific Islanders to BNT162b2 vaccination and Delta/Omicron infection: a cohort study' is exceptionally well-written. It provides evidence from Pacific communities that the Pfizer mRNA vaccine is effective in elucidating humoral immune response and ADCC among adults and elderly people although the outcomes shown to vary across age and by sex.

We are grateful to the reviewer for this very positive feedback on the value of our work.

However, I have the following few comments for authors to address before their manuscript is being considered for publication in PLOS Medicine.

-In addition to characterizing the humoral immune response of Pacific Islanders to the Pfizer BNT162b2 vaccine and SARS-CoV-2 infection, the study also investigated ADCC. I, therefore, suggest the authors consider modifying the title of their manuscript in such a way it also includes ADCC.

We agree with the reviewer that it is important to mention in the title this relevant and not frequently assessed feature of antibodies. We propose to indicate in the title that we assessed both the levels and the functionality of Pacific Islanders’ antibodies elicited by vaccination and natural infection. Functionality covers both antibodies’ neutralization capacity and their ability to mediate ADCC. We propose the following title:

“Levels and functionality of Pacific Islanders’ hybrid humoral immune response to BNT162b2 vaccination and Delta/Omicron infection: a cohort study in New Caledonia”

-Obesity is considered to be one of the comorbidities reported to be associated with developing symptomatic SARS-CoV-2 infection and severe COVID-19. Surprisingly, the current study observed that obese individuals (with a BMI > 30 kg/m²) had significantly higher anti-Spike IgG levels at one month post-immunization. This finding is intriguing and warrants further investigation. Perhaps obesity-related factors influence the immune response differently in this context. The authors shall provide possible explanations for this observation.

We agree with the reviewer that this interesting finding should be discussed: excessive presence of inflammatory adipose tissues in obese individuals may favor the establishment of a pro-inflammatory state favoring the early secretion of higher antibody titers. We added the following sentence to the Discussion Line 476-482 page 20: “Although excess adiposity is reported to negatively impact immune function and host defense in obese individuals^32^, presence of visceral pro-inflammatory adipose tissues ^33^, dysregulation in extracellular vesicles derived from adipose tissue and imbalance in the biosynthesis and levels of polyunsaturated fatty acid-derived oxylipins ^34^ as well as secretion of adipokines and hypoxia ^35^ in obese individuals maintain a basal pro-inflammatory state prone to activate immune cells, which may contribute to an enhanced immune response, including the early secretion of higher antibody levels.”

-Although the study evaluated anti-Nucleoprotein antibodies for identification of reinfection and breakthrough infection in Pacific Island cohorts, it did not directly compare titer levels across different ethnic groups. It would indeed be interesting to explore these differences further. In addition, the kinetics of anti-Nucleoprotein antibodies over time were not explicitly discussed in the study. Investigating the temporal changes in these antibodies could provide valuable insights.

Classification of participants into the “Vaccinated” and “Infected” groups was indeed based on the presence of anti-N IgG. Furthermore, for participants who underwent a longitudinal sampling, temporal changes in anti-N IgG levels allowed to define breakthrough infections and re-infections as follows:

-Breakthrough infection: anti-N IgG negative at the first sampling timepoint and anti-N IgG positive at the second sampling timepoint

-Re-infections: increase in the anti-N IgG levels between the first and the second sampling timepoint.

Stable or decreasing anti-N IgG levels between the two sampling timepoints were considered as the normal course of anti-N IgG levels following an infection before the first sampling timepoint. The decrease in anti-N IgG is probably highly dependent on the timing of the infection with regard to the first sampling timepoint, Despite the fact that the date of this infection was not documented in our cohort, we studied, as per the reviewer’s recommendation, (i) the levels of anti-N IgG across communities in participants at one month post-immunization and (ii) the decrease in anti-N IgG across communities in participants who were anti-N IgG positive at the first sampling timepoint and who did not show a sign of reinfection at the second sampling timepoint. Univariate linear regressions showed no difference between communities in the anti-N IgG levels at one month post-immunization. However, we observed a steeper decrease in anti-N IgG levels between one and six-months post-immunization in Melanesian participants, albeit in a reduced number of participants (9 Melanesian participants).

We therefore modified the Methods section line 219-223 page 10: “Furthermore, for participants who underwent a longitudinal sampling, breakthrough infections were defined as an anti-N IgG negative sample at the first sampling timepoint and an anti-N IgG positive sample at the second sampling timepoint; reinfections were defined based on an increase in the anti-N IgG levels between the first and the second sampling timepoint.”

We modified the results section line 370-373 page 16: “In participants who were anti-N IgG positive at D3.1, anti-N IgG levels were independent from the ethnic community (*p* value = 0.056, Supplementary table 8). Among those participants, in those who did not show signs of reinfection (decrease or stable anti-N IgG level) at D3.6, the decrease in anti-N IgG was steeper in the Melanesian community (9 participants, *p* value = 0.005, Supplementary table 9).”

We added to the discussion section line 446-448 page19: “Despite a steeper decrease in anti-N IgG over time in the Melanesian community, albeit in a reduced number of participants, we show that the humoral immune response to natural infection was overall similar across communities.”.

Reviewer #4:: This paper discusses immune response to vaccination and infection with SARS–CoV–2 in various communities.

I was asked for a methodological report and I interpret that to include all aspects of the design and conduct of the study. The authors have made the decision to depart from the usual order of sections in a scientific report except in the abstract. I think there is usually a good reason to follow the conventional order so I have arranged my comments accordingly.

We thank the reviewer for his thorough analysis of our work. We would like to mention that we re-arranged the sections: the Methods section is now after the Introduction and before the Results section.

**Points of detail**

*Summary and introduction*

Page 1 I would put New Caledonia in the title. Although there is a move to use vernacular geographical names in English I do not think we are ready for Nouvelle Calédonie yet sadly.

In order to take into consideration the reviewer’s recommendation as well as reviewer 3’s recommendation, we modified the title of our work as follows

“Levels and functionality of Pacific Islanders’ hybrid humoral immune response to BNT162b2 vaccination and Delta/Omicron infection: a cohort study in New Caledonia”

Page 3 Since the point of the paper is differences between ethnic groups I think we need more detail here about those differences or their absence and less about other covariates.

We agree with the reviewer that more emphasis should be put in the summary on the differences between the ethnic communities. We therefore modified the Summary Line 50-56 page 3 as follows: “At one-month post-immunization, the four ethnic communities exhibited no significant differences in their anti-Spike IgG levels (*p* value=0.17 in an univariate linear regression model), in their capacity to mediate Omicron neutralization (*p* values=0.59 and 0.60, in an univariate logistic regression model at one-month after the second and third dose, respectively) and in their capacity to mediate ADCC (*p* value = 0.069 in a multivariate linear regression model), regardless of the infection status. Anti-Spike IgG levels and functionalities of the hybrid humoral immune response remained equivalent across the four ethnic communities during follow-up and at six months post-third dose.”

Page 6 Why use both White and European here? Do the authors intend us to see them as different? The phrase ‘of European descent’ is often useful.

We apologize for this misleading denomination of people of European descent, used in reference 4. We rephrased the sentence line 104-107 page 6 as per the reviewer’s recommendation: “As vaccination protects from severe COVID-19 in cohorts mostly composed of people of European descent ^4^, it is pivotal to assess whether Pacific Islanders’ humoral response to anti-COVID-19 vaccination is equivalent to the one of people of European descent.”

*Methods*

- Page 14 I am afraid this section leaves me ignorant of many important details and confused about the authors’ intentions. The study is registered as a trial but there does not seem to be an intervention (which is not compulsory of course). How were people recruited into the study? Is this a consecutive sample of attenders at a routine clinic or were they invited to take part in a research project

“[The International Committee of Medical Journal Editors’(ICMJE)] requires, and recommends that all medical journal editors require registration of clinical trials in a public trials registry at or before the time of first patient enrollment as a condition of consideration for publication.” (<https://www.icmje.org/recommendations/browse/publishing-and-editorial-issues/clinical-trial-registration.html>). In our study, blood samples were taken from the participants for the sole purpose of the current research project and not in the framework of any intervention justified by their medical care. In this context, and as per the sponsor’s usual practice (the Institut Pasteur was the sponsor of this study), the study was registered in Clinical trials (ID: NCT05135585).

We apologize for not having clearly defined our intentions. Participants in this study were approached in vaccination centers, where they presented to receive their second or third dose of Pfizer BNT162b2 vaccine. Adult subjects were offered to participate in the current research project, which involved to provide blood samples at distance from the time of vaccination. In order to describe more accurately the study’s implementation, we added the Methods section line 160-161 page 8: “Participants presenting to immunization centers to receive a second or third dose of the Pfizer BNT162b2 were offered to participate in the current research project.”

- Page 15 We read ‘Not all participants enrolled in the study participated in the follow–up.’ Why? Loss to follow–up is important especially in an equivalence trial and needs to be explained somewhere.

Loss of follow-up was due to impossibility to contact participants, participants not presenting at follow-up visits or participants’ refusal to pursue their participation in the study. We added the following sentence to the Methods section line 179-182 page 9: “Due to impossibility to contact some participants, due to the absence of participants’ presentation at follow-up visits or due to some participants’ refusal to pursue their participation in the study, not all participants enrolled in the study participated to the follow-up as described in Figure 1.”

- Page 17 Why categorise age? Categorising an essentially continuous variable wastes information (Altman and Royston, 2006; Royston et al., 2006) and leads to models which are often implausible as they predict the effect remaining flat within categories and then jumping to a new value at the category boundary. For presentation it may be permissible but for modelling it is undesirable.

We thank the reviewer for this comment. We agree that age is a continuous variable, however it is unsure that the effect of age on IgG levels or on the odds of neutralization is linear. We could evaluate a non-linear effect by adding non-linear combination of the age (e.g. cubic splines), but then interpretation is not that straightforward. Moreover, recommendations for COVID-19 vaccination were by age categories, as the response is believed to differ in older populations. Currently, the French Health Authorities (Haute Autorité de Santé, HAS) indeed recommends a booster dose in individuals aged ≥65 years (https://www.has-sante.fr/jcms/p_3517088/fr/avis-n2024-0022/ac/sespev-du-11-avril-2024-du-college-de-la-haute-autorite-de-sante-relatif-a-l-edition-2024-du-calendrier-des-vaccinations). We chose to categorize age to investigate if we could detect an indication of worse immune response in older subjects. We found that older individuals aged ≥65 years exhibited lower antibody levels after two doses of BNT162b2. We also found that individuals aged ≥40 years had lower odds of Omicron neutralization at D3.6.

Page 17 Selecting a subset of variables in a way driven by the data leads to a model which is unlikely to replicate (Babyak, 2004). Screening variables to enter the model has been criticised too (Sun et al., 1996) What was the scientific question which made selection necessary?

The goal of these analyses was to identify factors that were associated with the level of the immune response on the one hand, and with the neutralization capacity and capacity to mediate ADCC on the other hand. We did not intend to build a score to predict good response to vaccination, but rather described which factors were associated with the response. In that sense, the factors that presented a high not-significant *p*-value in univariate analysis were clearly not associated with the outcomes of interest, justifying not to consider them in the multivariate analysis, which is common practice in epidemiological studies.

*Results*

Page 8 According to the methods (page 14) the primary outcome was the fraction exhibiting an anti–S IgG response. I have searched here and I do not find this reported. We do get an analysis of levels but that is not the same.

We thank the reviewer for pointing out this missing information. We found that all participants developed anti-S IgG in response to vaccination, even as early as 1 month after the second immunization. As a consequence, studying the proportion of participants that presented anti-S IgG was no longer relevant. We modified the Results section line 299-301 page 13: “Anti-S IgG were detected in all samples at all timepoints. Therefore, the planned analysis of the fraction of participants presenting anti-S IgG was no longer relevant. We investigated the levels of anti-S IgG, the neutralization capacity and the capacity to mediate ADCC.”

Page 8 The ROC analysis does not seem to be described in the methods. It is not clear to me what it is telling me.

We apologize for this missing information. The ROC analysis is now described in the Methods section line 267-269 page 12: “A ROC (Receiver Operating Characteristic) curve analysis was used to identify the anti-S IgG threshold that best discriminated participants able to neutralize Omicron with an optimal sensitivity and specificity”

In four pages of results we only seem to get six sentences about the differences between communities and those are rather bald. Is there some reason for this emphasis or lack of it?

We measured the impact of the ethnic community for all the parameters studied and at all timepoints. We found that the level of anti-S IgG, the neutralization capacity and the capacity to mediate ADCC did not differ between communities in our study, suggesting that the response to vaccine was similar between the communities. This important result was stated in the Results section and was also part of the Discussion section. We added the following sentence in the Discussion section line 513-520 page 21-22 to emphasis the importance of this result: “This study has important implications: evidence of a robust and prototypical humoral immune response to Pfizer BNT162b2 vaccine and SARS-CoV-2 in Pacific Islanders is of prime importance to foster booster doses roll-out, with the aim to better protect this under-studied population, at risk for severe COVID-19. Furthermore, as obesity is highly prevalent in Pacific populations and is associated with higher anti-Spike antibody levels, results from this study will contribute to adapted vaccination regimens, including booster doses roll-out, to better protect Pacific Islanders.”

*Discussion*

Page 13 The term metis was not familiar to me. Investigating in Wikipedia the specific entries for it seem to suggest it is used differently in English, where it seems to refer primarily to quite specific communities in the US and Canada, and in French, where métis is equivalent to the English term mixed race or one of its variants. Wikipedia might be wrong of course but it might be better to use an alternative term.

We thank the reviewer for this accurate definition of the term “metis” in English. We modified in the text of the Discussion Line 499-500 page 21 “Although mixed marriages are common, participants had the opportunity to identify as “Other communities”, which included people of mixed race.”

Page 13 The authors state as a limitation ‘. . . finally the sample size was not reached, hindering to ascertain response equivalence between communities.’ No, the reverse is the case. Under–recruitment in an equivalence trial biases towards finding equivalence (Jones et al., 1996).

As specified in the Results section, the sample size was not reached in the Polynesian community. We therefore modified this sentence in the discussion section line 507-512 pages 21: « Finally, the sample size was not reached for the Polynesian community. Under-recruitment tends to bias the results towards non significance; therefore, one cannot exclude that this could explain the similar responses to vaccination we observed in the different communities.”

**Points of more substance**

*Representativeness*

I have already remarked that we do not know how people were recruited into the study. This is made more salient since the participants do not appear with the population frequencies given on page 6. This is rather obvious but for the record a formal test gives a χ 2 3 = 63.9 with associated probability way beyond any conventional α level (p = 8.5 × 10−14). Is this because there was some form of stratified sampling?

The reviewer is indeed right: as the study was designed as an equivalence study, we targeted to enroll 141 participants in each community, which is decorrelated from the proportion of each community in New Caledonia’s general population. We therefore performed a stratified sampling. In order to specify it, we modified the Methods section line 172-174 page 8 as follows “A stratified sampling was thus conducted in order to enroll 141 participants in each of the Melanesian, European, Polynesian and “Other” community.”

*Width of confidence intervals*

The authors had a target of 141 per group (page 14). The actual numbers are much lower and I calculate that, all else being equal, the width of the confidence intervals has been inflated by about 1.35 times.

In my table 1 I show what would happen if the standard errors had been deflated by a factor of 1.35. As can be seen this alters the conclusions. I am not saying that if the design sample size had been achieved the confidence intervas would have been as my table shows but I do think that we need much more caution here about any attempt to claim that the various communities had the same response.

We thank the reviewer for this comment. As answered to one of his/her previous comment regarding the conclusion, we modified the discussion to present the fact that under-recruitment tends to favor the conclusion in favor of non-significance. The discussion line 507-509 pages 20 now reads « Finally, the sample size was not reached for the Polynesian community. Under-recruitment tends to bias the results towards non significance; therefore, one cannot exclude that this could explain the similar responses to vaccination we observed in the different communities.”

*Is the other category relevant?*

The other category of ethnicity is presumably a mixture of people who refused to say, people who identify as another category, and people of mixed race. It is hard to see them being relevant to the primary issue here, whether Pacific Islanders have a different response to people of European descent. I suggest removing them and only including them in a sensitivity analysis.

New Caledonia’s population is highly diverse and mixed: as much as 26.5% of the general population self-declares as belonging to other communities, probably including some people who refuse to self-identify as belonging to a specific community and, most importantly, 11.3% of people of mixed race (ISEE-NC). Implementation of this study in New Caledonia was highly conditioned by population acceptance and community engagement. In this context, it was pivotal to implement an inclusive study in which any inhabitant of New Caledonia could participate, provided that he/she met the eligibility criteria, and most importantly, regardless of his/her ethnic community. We recognize that, from a biological and genetic perspective, this group is not representative of any specific genetic ancestry, but, for the sake of representativity and with the aim that New Caledonia population takes ownership and benefits from the results of this study, we will keep this group in the overall presentation of the results. However, complementary analyses, in which we did not consider the “Other communities” group, were performed at one-month post-immunization. They can be found in Supplementary tables 3-5. They show a lack of difference between the Melanesian, European and Polynesian communities in terms of anti-S IgG levels, capacity to neutralize Omicron and capacity to mediate ADCC.

We added to the methods section line 281-282 page 12-13 “Sensitivity analyses not considering participants self-declaring as belonging to other communities were performed at one-month post immunization.”.

We added the following text in the results section line 355-358 page 15: “Sensitivity analyses not considering the “Other communities” group performed at one-month post immunization confirmed our initial findings regarding the lack of difference between the Melanesian, European and Polynesian communities in terms of anti-S IgG levels, capacity to neutralize Omicron and capacity to mediate ADCC (Supplementary tables 3-5).”

We also discuss this methodologic choice in the limitations section of the discussion line 501-507 page 21: “The “Other communities” group was thus, as per definition, a highly diverse group of participants rich from its various genetic ancestries. Community engagement and population acceptance were paramount for both the successful implementation of the current study and subsequent ownership taking of the results by New Caledonia’s population. The current study was therefore inclusive and enrolled any participant regardless of her/his self-declared ethnicity, including people of non-European, non-Melanesian or non-Polynesian ancestry.”

*Summary*

I have raised a number of points which I think need clarifying and where details are currently in the authors’ heads rather than on the paper. It is unlikely that many similar data sets will be available and so I would not regard the failure to achieve the planned sample size as a fatal flaw but I do think it needs to be interpreted properly.
