# Supplementary material for: Levels and functionality of Pacific Islanders’ hybrid humoral immune response to BNT162b2 vaccination and delta/omicron infection: A cohort study in New Caledonia
Source: PLoS Med. 2024 Sep 26;21(9):e1004397. doi: 10.1371/journal.pmed.1004397 (PMC11466435; doi:10.1371/journal.pmed.1004397)
Supplement: Attachment — Submitted filename: COVCAL Response to reviewers20240731.docx [file pmed.1004397.s024.docx]

Dr Catherine Inizan

Institut Pasteur in New Caledonia

11, ave Paul Doumer

BP 61, 98845 NOUMEA Cedex

NOUVELLE-CALEDONIE

Tel +687 272666

cinizan@pasteur.nc

Nouméa, the 31^st^ of July, 2024

Dear Reviewers,

Please find below a point-by-point response to your comments on our manuscript entitled “**Levels and functionality of Pacific Islanders’ hybrid humoral immune response to BNT162b2 vaccination and delta/omicron infection: a cohort study in New Caledonia**" (PMEDICINE-D-24-01093R2).

We thank you for reviewing our manuscript. You have raised a number of complementary points that needed to be taken care of in order to improve the manuscript. More specifically, we have made substantial changes to the material and methods section, which now describes exhaustively the study design. We propose here a point-by-point response to your comments, describing any additional analyses that were carried out, and propose a revised version of the manuscript.

We have ensured that all changes to the manuscript are indicated in the text by using track changes.

We hope this revised version will meet your recommendations and suggestions for improvement.

Sincerely yours,

Dr Catherine Inizan, on behalf of all authors.

Comments from the editorial team:

Thank you again for submitting this revised manuscript. We continue to find your study interesting, but are concerned about the level of missingness of key information on how the study was conducted. We are in agreement with the points raised by the reviewers and, especially, the statistician (Reviewer 4), and ask that you address all points in full. Given the significance of the aforementioned issues, we note that not addressing these concerns sufficiently would likely result in the journal choosing not to take your manuscript any further.

We thank the editorial team for its interest in our manuscript. We understand that key information was missing to fully understand how the study was conducted. We have made substantial changes to the material and methods section describing the study design and have amended Figure 1.

In addition to the above, we have listed some smaller points for you to address below:

(1) It is our understanding that you detected anti-spike antibodies in 100% of participants – please report this in your abstract.

We have added this important information to the abstract line 48 page 3.

(2) We see no need to abbreviate New Caledonia to NC; we would prefer if you would be so kind to spell it out throughout.

We have replaced any occurrence of NC to “New Caledonia”, as per the editor’s recommendation.

(3) We ask that ‘nucleoprotein,’ ‘spike,’ ‘glycoprotein’ (etc) not be capitalized, nor SARS-CoV-2 variants (e.g. alpha, delta, omicron - Wuhan is an exception).

We have modified the spelling of “nucleoprotein”, “spike”, “glycoprotein”, “delta”, and “omicron” to remove the capital letters.

(4) Line 125 - please remove the word ‘deployed’ and revise the sentence so that it reads ‘Humoral and cellular responses among Australian First Nations people were similar…’ or something similar.

We thank the editor for this suggestion and rephrased this sentence. The manuscript line 121-123 page 7 now reads “Similarly, humoral and cellular responses to Pfizer BNT162b2 vaccine was similar in Australian First Nations and Non-Indigenous people ^12^.”.

(5) Lines 138-140: ‘We aimed to characterize Pacific Islanders’ hybrid humoral response to BNT162b2 vaccine and SARS-CoV-2 infection, by evaluating anti-Spike (anti-S) IgG levels, the neutralization capacity…’ – please also mention anti-nucleocapsid antibodies in this sentence.

We thank the editor for this suggestion and added this information to the sentence. The manuscript line 135-139 page 7 now reads “We aimed to characterize Pacific Islanders’ hybrid humoral response to BNT162b2 vaccine and SARS-CoV-2 infection, by evaluating anti-nucleoprotein IgG levels, anti-spike (anti-S) IgG levels, the neutralization capacity and the ability to mediate ADCC of sera longitudinally collected in a cohort of 585 vaccinated adults from New Caledonia self-declaring as Melanesians, Polynesians, Europeans or belonging to other communities.”

Comments from the academic editor:

The Academic Editor agreed with the comments raised by the statistical reviewer and believed these were important to address. He supported the decision to send your manuscript back to you for a further revision.

We thank the Academic Editor for giving us the opportunity to improve our manuscript.

-----------------------------------------------------------
Comments from the reviewers:

Reviewer #1: I thank the authors for attending to my previous comments so comprehensively. There are just a couple of comments.

1. Whilst the authors have included a sentence in the methods section line 164-166 page 8 clarifying the inclusion criteria for timings between doses. It would be useful to include the mean and range between each dose, as this is important information needed when interpreting the data.

We added to the text of the results section line 296-298 page 13:

“Median duration between the first and second dose was 21 days (inter-quartile range [21, 22]) and median duration between the second and the third dose was 147 days (inter-quartile range [128; 183]).”

2. The paper by Hartley et al. shows SARS-CoV-2-specific IgG levels in individuals plasma. This is quantified to different SARS-CoV-2 strains hence the query to whether this had been quantified e.g. in ELISA.

We understand the reviewer’s point and that others have developed ELISA assays to quantify the levels of anti-SARS-CoV-2 IgG targeted at different variants. In our case, the LuLISA technique we used did not allow us to quantify variant-specific IgG levels, although we agree that this is of interest. Instead, we quantified the IgG levels against the ancestral Wuhan strain and assessed the sera neutralizing capacity against the ancestral strain and the delta, omicron BA.1 and omicron BA.4/5 variants.

We added this limit to the discussion section line 505-508 page 22:

“Although the LuLISA assay quantified total anti-spike IgG levels and not variant-specific IgG levels, the pseudoneutralization assays allowed to discriminate sera containing variant-specific IgG levels above the neutralizing threshold.”

3. For neutralisation, please specify the change in neutralisation levels between doses, so that it is clear whether this changes between doses. Also, specify % patients with positive Spike-specific IgG to show whether seroconversion levels change between doses.

In our pseudovirus neutralization assay, we qualitatively assessed the capacity of each serum sample to neutralize each variant: instead of neutralization levels, we measured the capacity (“yes/no”) of a single dilution (1/40) of each of our 849 serum samples to neutralize the 4 viral strains tested. Therefore, we cannot provide a neutralization level nor a change in this neutralization level between doses. We nonetheless observe a very significant increase in the number of sera exhibiting the capacity to neutralize omicron variant between the second and the third dose. To make our point clearer, we rephrased Line 329-332 page 15:

Levels of anti-S IgG were significantly higher (*p*<0.001, Table 1 and Supplementary Table 2) and the ability to neutralize omicron was significantly more frequent (*p*<0.001, Supplementary Table 2) one month after the third dose than after the second dose, irrespective of the infection status (Figure 3a).

And Line 350-353 page 15:

The ability to neutralize omicron BA.1 and/or BA.4/5 however significantly differed by number of doses (Figure 3C): the proportion of participants neutralizing omicron increased from 19.1% after the second dose to 97.9% after the third dose (Supplementary Table 2).

Also, we want to draw the reviewer’s attention on the fact that at all timepoints studied, 100% of the serum samples contained anti-spike IgG. It is written line 310 Page 14 and added to the abstract line 47 page 3 as per the editor’s recommendation.

Reviewer #3: I have no further comments/remarks. All the comments and remarks I made in my previous review were appropriately addressed.

We thank the reviewer for his/her appraisal of our answers to his/her review.

Reviewer #4:

This paper discusses immune response to vaccination and infection with SARS–CoV–2 in various communities. I was asked for a methodological report and I interpret that to include all aspects of the design and conduct of the study

Points of detail

I said:

*We read ‘Not all participants enrolled in the study participated in the follow–up. Why? Loss to follow–up is important especially in an equivalence trial and needs to be explained somewhere.*

Figure 1 does contain some information as stated in the rebuttal but it does not specify the reasons for people not appearing at the various stages. How many refused to be recruited? How many became untraceable?

We did not record the number of vaccinees who were informed of the research project and refused to take part in the study. We recognize that this metrics would be a fair estimate of population’s acceptance of the study and will make sure to collect this data in future population studies. All participants “pre-included” in the second and third dose cohorts accepted to be recruited: they filled a consent form, answered to the questionnaire and their personal details were collected. One month after immunization, three months after the second dose and six months after the third dose however, up to 25.4% of the participants were not sampled, either because they clearly expressed their will to be excluded from the study (5.6%) or because they could not be contacted or did not present at the follow-up visit (19.9%). We added the following sentence to the results section Line 304-305 page 14:

Overall, 38 (5.6%) participants refused to pursue their participation in the study and 135 (19.9%) participants were untraceable (Figure 1).

We also adjusted Figure 1 to better specify the three inclusion periods, the number of participants not followed-up and the number of participants who discontinued their participation or became untraceable.

Figure 1:

I said:

*Selecting a subset of variables in a way driven by the data leads to a model which is unlikely to replicate (Babyak, 2004). Screening variables to enter the model has been criticised too (Sun et al., 1996) What was the scientific question which made selection necessary?*

I did not find the authors’ rebuttal not very convincing here. Just because a variable does not reach some arbitrary level of statistical significance in a univariate analysis does not mean it will or will not contribute in the multivariable one. If the univariate analyses could tell us what the multivariable analysis was going to say we would not have needed to invent multiple regression. Why not present a full model with all covariates which might be important?

As per the reviewer’s recommendation, we included all variables in the multivariate models presented in the main and supplementary tables, even those reaching a *p* value >0.2 in the univariate analyses. Results from these complementary analyses were added to the tables. We observed that for all outcomes studied and at all timepoint, variables found significantly associated with the outcome in the multivariate model including all variables remained significantly associated with this characteristic in the multivariate model built using the backward stepwise procedure. Presenting a simplified model allows to better assess the relative contribution of a reduced number of variables significantly associated with the characteristic of interest. We therefore decided to show the results of both multivariate models. We added to the Methods section line 279-281 page 13:

A multivariate model including all variables was built. Subsequently, a backward stepwise procedure was used to identify factors that remained independently associated with the outcome.

The three main tables and all relevant supplementary tables have been modified accordingly.

I said:

*The ROC analysis does not seem to be described in the methods. It is not clear to me what it is telling me.*

What motivated the choice of cut–off for sensitivity and specificity? On the face of it missing true cases is more important that picking false positives so the present choice needs justifying.

The ROC curve helped us identify the IgG level that best classified the patients able and not able to neutralize omicron. In the method section, we mentioned “A ROC (Receiver Operating Characteristic) curve analysis was used to identify the anti-S IgG threshold that best discriminated participants able to neutralize Omicron with an optimal sensitivity and specificity.” Although our sentence could be understood as giving as much importance to sensitivity and specificity, we want to emphasize that we favored specificity. We modified the sentence that now reads (Line 269-272 page 12): “A ROC (Receiver Operating Characteristic) curve analysis was used to identify the anti-S IgG threshold that best discriminated participants able to neutralize omicron with an optimal specificity.”, we added to the results section Line 343-347 page 15 “In a ROC curve analysis, a threshold of 5.737 AU maximized the proportion of subjects correctly classified in terms of neutralization capacity, but importantly the threshold maximized the capacity to identify those unable to neutralize omicron (57 out of 60, 95% specificity)” and added a sensitivity/specificity table to the supplementary figure 3.

If it is important to identify the threshold above which patients can neutralize omicron, it is more important to us to correctly identify those who are unable to neutralize omicron, thus giving priority to specificity. Indeed, identifying those who could benefit from vaccine booster could be of importance for public health policy. Accordingly, we added to the discussion section Line 454-457 page 20: “We evidenced that anti-S IgG levels above 5.737 AU in LuLISA assay discriminate with 95% specificity the participants able to neutralize omicron, providing useful decision-aiding tool to accurately identify those likely to benefit from a booster dose.”

I said:

*I have already remarked that we do not know how people were recruited into the study. This is made more salient since the participants do not appear with the population frequencies given on page 6. This is rather obvious but for the record a formal test gives a χ 2 3 = 63.9 with associated probability way beyond any conventional α level (p = 8.5 × 10−14). Is this because there was some form of stratified sampling?*

In their rebuttal the authors mention stratified sampling. How exactly was this managed? When people presented for their vaccination were their details taken and then a decision made as to eligibility? Or did their medical records already have information on their self–reported ethnic group? Did the central office monitor recruitment and then issue an order to stop recruiting from that ethnicity when 141 had been recruited? Note that if this means that the Polynesian group were still being recruited when all the other ethnicities had stopped this is not best practice if there might be secular effects.

The sampling strategy implemented in the current study was based on a weekly monitoring of the number of participants enrolled in each community. Representativeness of the four ethnic communities on the entire inclusion period was ensured through targeted enrolment in health centers where the vaccinated population was reportedly belonging to each of the communities of interest. Distribution of the four ethnic communities indeed exhibits a geographical partitioning at the scale of the municipalities and in the different districts (ISEE, 2019 census). Distribution of the four ethnic communities according to the enrolment date demonstrates that the four ethnic communities were equally distributed along the inclusion period. Inclusion of a reduced number of Polynesian participants results from the lowest number of Polynesians among New Caledonia population (8.3%) and the reduced vaccination rate in this community.

We modified the methods section Line 149-193 pages 8-9 to better explain how we implemented the study, and gave more details as to how the representativity of the four communities was ensured:

*Cohort and study design*

The current study follows the STROBE (**STrengthening the Reporting of OBservational studies in Epidemiology)** reporting guidelines (<https://www.strobe-statement.org/>). The filled STROBE checklist is shown in Supplementary materials. Enrolment of adults (≥18 years) vaccinated with two or three doses of the Pfizer BNT162b2 vaccine took place in three phases in immunization and health centers in the South Province of New Caledonia (Supplementary figure 1). Between December 2021 and January 2022, adults who received the second dose of the Pfizer BNT162b2 vaccine were enrolled (Figure 1A). Between January 2022 and March 2022, when information campaigns largely promoted immunization with a third dose, adults who received a third dose were enrolled (Participants enrolled at the third dose, Figure 1B). Enrolment in immunization centers took place every working day to maximize the number of enrolled participants at the peak of vaccination roll-out. Between March and August 2022, when the number of third doses delivered decreased, individuals having received a third dose within the last 6 months were enrolled through targeted information campaigns (Participants enrolled remotely from the third dose, Figure 1B). Sample size calculation for this equivalence study was based on a power of 85% to detect changes of more than 5% in the fraction of participants developing anti-S IgG in response to Pfizer BNT162b2 vaccination, with an alpha risk of 5%, resulting in the target to enroll 141 participants in each of the Melanesian, European, Polynesian and “Other” community. Based on a weekly monitoring of the number of participants enrolled in each of the four communities, enrolment was targeted in immunization and health centers located in specific geographical areas mostly populated by the communities of interest (ISEE, 2019 census). Individuals fulfilling inclusion/exclusion criteria were presented the research project by a member of the investigation team; they were given time to read the information notice and decide whether they wanted to participate in the study. During the inclusion visit, inclusion criteria were verified: having received the second dose between three and twelve weeks after the first dose, and the third dose between three and six months after the second dose were inclusion criteria. Documented SARS-CoV-2 infection at any time before inclusion (serologic, PCR or antigenic testing) was an exclusion criterion. Pregnant or breast-feeding women, immunosuppressed individuals, individuals unable to answer the questionnaire and those under protective measures were excluded. Following signature of the consent form, clinico-epidemiological data (age, self-declared gender, self-declared ethnicity, medical history, height and weight) were recorded in REDCap (Vanderbilt University, USA). Personal contact details were collected to organize the follow-up visits.

For all participants, follow-up visits were to be performed one month (+/- 10 days) and 3 months after the second dose or one month (+/- 10 days) and/or 6 months after the third dose. The week preceding the planed sampling period, the investigation team arranged a home visit by a qualified nurse by contacting all participants by phone, adapting as best as possible to participants’ availability. Upon these visits, a 4-mL venous blood sample was collected. Sera were separated from blood samples by centrifugation and stored at -20°C until subsequent analyses.

Sampling took place between January and August 2022. Not all participants enrolled in the study participated to the follow-up: some participants became untraceable (impossibility to contact the participants, absence of presentation at follow-up visits), others refused to pursue their participation in the study (expressed during phone contacts), as shown in Figure 1.

And to the text of the results section line 290-294 page 13:

The study period was short, from December 2021 to August 2022, thus limiting the risk of selection bias between communities. As the targeted sample size for each community was not reached in either of the second- and third-dose cohorts, participants from all four communities were enrolled along the entire enrolment period (Supplementary figure 2).

Supplementary figure 2:

Conclusion

As I remarked in my original review this is a data–set which is unlikely to be repeated and if the clinical reviewers feel it is an important study then we can take a more relaxed attitude to any problems I feel I have identified. Nonetheless I think for transparency we do need more details, as I have outlined.

We thank the reviewer for his request to give more details on the study design, as this investigation work was pivotal for the proper implementation of the reported research.
