# Supplementary material for: Levels and functionality of Pacific Islanders’ hybrid humoral immune response to BNT162b2 vaccination and delta/omicron infection: A cohort study in New Caledonia
Source: PLoS Med. 2024 Sep 26;21(9):e1004397. doi: 10.1371/journal.pmed.1004397 (PMC11466435; doi:10.1371/journal.pmed.1004397)
Supplement: Attachment — Submitted filename: Response to Reviewers 20240830.docx [file pmed.1004397.s025.docx]

Dr Catherine Inizan

Institut Pasteur in New Caledonia

11, ave Paul Doumer

BP 61, 98845 NOUMEA Cedex

NOUVELLE-CALEDONIE

Tel +687 272666

cinizan@pasteur.nc

Nouméa, the 30^th^ of August, 2024

Dear Reviewer,

We warmly thank you for your comments and suggestions on our manuscript entitled “**Levels and functionality of Pacific Islanders’ hybrid humoral immune response to BNT162b2 vaccination and delta/omicron infection: a cohort study in New Caledonia**" (PMEDICINE-D-24-01093R3).

Please find herewith a revised version of our manuscript taking into consideration your comments. You will find below a point-by-point response to those comments, describing any additional analyses and modifications to figure 3 that were carried out.

We have ensured that all changes to the manuscript are indicated in the text by using track changes.

We hope this revised version will meet your recommendations and suggestions for improvement.

Sincerely yours,

Dr Catherine Inizan, on behalf of all authors.

EDITORIAL COMMENTS:
Thank you again for engaging so thoroughly with the reviewer and editorial comments. We appreciate the value of the data presented here and are confident that this will make an impact on an underrepresented population.

As noted earlier in this email, Reviewer 1 made a number of points. We consulted the academic editor on this review and he asked that you kindly address 2 of the reviewer’s comments (see below). He believed that the changes required would be unlikely to make a change to the overall conclusions and, hence, supported a minor revision outcome at this stage.

The academic editor recommended that the authors address the following:
(1)     ‘It has been shown in numerous publications that neutralisation of Omicron BA.1 and BA.4/5 differ both at post dose 2 and post dose 3 of COVID-19 vaccination. In lines 350-355 the authors provide a blanket statement that neutralisation of omicron increases from 19.1% at dose 2 to 97.9 at dose 3. Which omicron variant is this to and are there differences in neutralisation of each variant as has been previously published.’ He asked that some comment/clarification be provided here, with no need for further experiments, etc.

We thank the reviewer for raising this interesting point. As stated in the methods section (Line 241-242 page 11), in the current manuscript, omicron neutralization is defined as a “≥ 90% neutralization of either omicron BA.1 and/or omicron BA.4/5 pseudoviruses in pseudovirus neutralization assays (i.e. neutralization titer >40)”. We added to the text line 348-356 page 16 “When considered separately, 17.6% (12/68) and 11.8% (8/68) of the participants were able to neutralize omicron BA.1 and omicron BA.4/5 at one month post-second dose, respectively. In contrast, 97.0% (230/237) and 93.7% (222/237) of the participants were able to neutralize omicron BA.1 and omicron BA.4/5 at one month post-third dose, respectively. For both variants, the proportion of neutralizing participants increased significantly between the two doses (*p* <0.0001, Chi^2^ test). In addition, the proportion of participants neutralizing omicron BA.1 was not significantly different than the proportion of participants neutralizing omicron BA.4/5, neither at one month post-second dose (*p* = 0.33) nor at one month after the third dose (*p* = 0.08).”

(2)  ‘Figure 3C is not easy to understand and why does the scale for % neutralisation only go up to 1 when you report in the text that the neutralisation went up to 97.9 at dose 3. I would suggest graphing this in a different way to make it easier to understand.’

We want to draw the reviewer’s attention on the fact that 97.9% refers to the percentage of participants exhibiting a ≥90% neutralization of omicron BA.1 and/or omicron BA.4/5 out of all participants sampled at one month post-third dose, and not the absolute percentage of neutralization for each participant. The text Line 347-348 page 16 indeed states “the proportion of participants neutralizing omicron increased from 19.1% after the second dose to 97.9% after the third dose (Supplementary Table 2)”. We modified the Figure 3C, which does not show the different percentages of neutralization on a 0 to 1 scale anymore. We rather show only the two categories defined for the variable “neutralization”: “no neutralization” in dark blue, corresponding to participants exhibiting less than 90% of neutralization of the considered variant, and “neutralization” in yellow, denoting participants with at least 90% of neutralization of the considered variant.

REVIEWER COMMENTS:
Reviewer #1: Whilst the authors have attended to most of the comments I posed, there are a still a number of outstanding issues that require attention prior to publication.

- It has been shown in numerous publications that neutralisation of Omicron BA.1 and BA.4/5 differ both at post dose 2 and post dose 3 of COVID-19 vaccination. In lines 350-355 the authors provide a blanket
statement that neutralisation of omicron increases from 19.1% at dose 2 to 97.9 at dose 3. Which omicron variant is this too and are there differences in neutralisation of each variant as has been previously published.
The answer to this comment can be read above.

-Is there any information of infection status of the subjects at each dose as this can have an impact on the level of anti-spike IgG and neutralisation titers measured in the serum. If not this should be stated. Did infection have an impact on the magnitude of response observed.

While the history of previous SARS-CoV-2 infection is documented through anti-N IgG detection at one-month post-immunization, the serostatus of participants was unknown at the time of immunization. We detected higher anti-S IgG titers, higher levels of ADCC and a higher proportion of omicron neutralization in infected participants as compared to non-infected participants, whether infection occurred prior to immunization or between immunization and participants’ sampling. In order to specify this information, we modified the text of the results section Line 309-313 page 15 as follows “Eighty-five participants (14.5%) reported a positive COVID-19 test at one of the sampling timepoints. Participants exhibiting anti-N IgGs, at D2.1, D2.3, D3.1 and D3.6, were 47 (69.1%), 43 (76.8%), 150 (63.3%) and 280 (57.4%), respectively (Figure 2). Detection of anti-N IgG evidenced a previous or current SARS-CoV-2 infection, whether it occurred prior to immunization or between immunization and sampling.”

- Figure 3C is not easy to understand and why does the scale for % neutralisation only go up to 1 when you report in the text that the neutralisation went up to 97.9 at dose 3. I would suggest graphing this in a different way to make it easier to understand.
The answer to this comment can be read above.

Reviewer #4: The authors' rebuttal has addressed my concerns.

EDITORIAL REQUESTS:
Abstract: Please combine the Methods and Findings section into one section with the heading ‘Methods and Findings’.

We combined both sections as per the reviewer’s recommendation.

Data Availability Statement: Are you able to provide an email address that is not for the corresponding author but perhaps to a generic inbox? Directing data requests to a non-author institutional point of contact, such as a data access or ethics committee, helps guarantee long term stability and availability of data. Providing interested researchers with a durable point of contact ensures data will be accessible even if an author changes email addresses, institutions, or becomes unavailable to answer requests.

As per the editor recommendation, we provided in the data availability statement the generic email address for clinical studies in our institution. The statement Line 582-584 page 26 now reads “Further information and requests for reagents, resources and data should be directed to and will be fulfilled by the department in charge of clinical investigations at the Institut Pasteur in New Caledonia, (ipnc-etudeclinique@pasteur.fr). Deidentified datasets are provided as Supplementary material.”

Author Summary:

(1) Could you expand the first sentence for our readers please? ‘Pacific Islanders are at risk for severe COVID-19’ – could you add a note here to explain why this is the case for the non-specialist reader?

Author summary Line 65-66 page 5 now reads “Pacific Islanders are at risk for severe COVID-19, in part owing to lifestyle and high rate of comorbidities.”

(2)     In the sentence ‘Characterizing the levels and functionality of Pacific Islanders humoral immune response to Pfizer BNT162b2 vaccine is pivotal to adapt vaccination regimen to regional singularities’ – I suggest changing ‘regimen’ to ‘regimens’ and adding a full stop at the end of the sentence.

We modified the sentence according to the editor’s recommendation.

(3)     In the sentence ‘The current study enrolled 585 participants from New Caledonia self-declaring as Melanesians, Polynesians, Europeans or belonging to Other Communities and characterized their antibodies levels and functionality at one and three months post second dose or one and six months post-third dose of the Pfizer BNT162b2 vaccine’, it may be best to break this up a little to improve readability. Perhaps something like this (or similar): ‘The current study enrolled 585 participants from New Caledonia self-declaring as Melanesians, Polynesians, Europeans or belonging to Other Communities. Antibody levels and their functionality were characterized at the following time-points: (1) one and/or three months after a second dose of the Pfizer BNT162b2 vaccine was administered, or (2) one and/or six months after the third dose of the vaccine.’

We modified the sentence according to the editor’s recommendation.

(4)     Perhaps include the word ‘all’ before ‘sera’ in the sentence ‘61.3% of the sera contained traces of previous SARS-CoV-2 infection; the cohort’s immune response was thus mostly hybrid, resulting from both vaccination and infection’.

We modified the sentence according to the editor’s recommendation.

(5)     As the Author Summary is meant to be non-technical and accessible to a non-expert audience, please revise the following sentence by explaining (briefly) what the relevance of ‘anti-spike IgG levels’ are and also explain the abbreviation ‘ADCC’. The sentence in question is: ‘At all timepoints analyzed, the hybrid humoral immune response remained equivalent across the Melanesian, Polynesian, European and Other communities: anti-spike IgG levels, capacity to mediate omicron neutralization and capacity to mediate ADCC were not significantly different across communities (p values>0.05).’

We modified the sentence Line 83-88 as follows “At all timepoints analyzed, the hybrid humoral immune response remained equivalent across the Melanesian, Polynesian, European and Other communities: anti-spike antibody levels, which mirror the strength of the immune response, but also the capacity to mediate omicron neutralization and the capacity to mediate Antibody-Dependent Cellular Cytotoxicity were not significantly different across communities (*p* values>0.05).”

Introduction:
Line 109: ‘Considering ethnic representation in COVID-19 vaccine trials is crucial.’ It might be useful to expand on this sentence so it matches what you eventually write in the Author Summary.

We modified the sentence Line 115-116 page 7 as follows “Considering ethnic representation in COVID-19 vaccine trials is crucial ^9^ to adapt vaccination regimens to regional singularities.”

Discussion:
Line 493-495: ‘Second, the high rate of SARS-CoV-2 infections prevented to assess ethnicity’s impact…’ – would this be better written as ‘…prevented us from being able to assess…’ or something of that ilk? Forgive me if I’ve misinterpreted the meaning behind this sentence.

The editor understood correctly what we aimed to mean. We therefore modified the sentence as per her/his suggestion.

Line 522: Please replace the semi-colon with a comma.

We replaced the semi-colon by a comma as recommended by the editor.
